# Omadacycline Efficacy against Enterococcus faecalis Isolated in China: *In Vitro* Activity, Heteroresistance, and Resistance Mechanisms

**DOI:** 10.1128/AAC.02097-19

**Published:** 2020-02-21

**Authors:** Zhiwei Lin, Zhangya Pu, Guangjian Xu, Bing Bai, Zhong Chen, Xiang Sun, Jinxin Zheng, Peiyu Li, Di Qu, Qiwen Deng, Zhijian Yu

**Affiliations:** a Department of Infectious Diseases and Shenzhen Key Laboratory for Endogenous Infections, Affiliated Shenzhen Sixth Hospital of Guangdong Medical University, Shenzhen, China; b Quality Control Center of Hospital Infection Management of Shenzhen, Shenzhen Nanshan People’s Hospital and the 6th Affiliated Hospital of Shenzhen University Health Science Center, Shenzhen, China; c Department of Infectious Diseases, Key Laboratory of Viral Hepatitis of Hunan Province, Xiangya Hospital, Central South University, Changsha, Hunan Province, China; d Key Laboratory of Medical Molecular Virology of Ministries of Education and Health, School of Basic Medical Science and Institutes of Biomedical Sciences, Shanghai Medical College of Fudan University, Shanghai, China

**Keywords:** omadacycline, *Enterococcus faecalis*, heteroresistance, multilocus sequence typing, tetracycline-specific resistance genes

## Abstract

This study aimed to evaluate the *in vitro* antimicrobial activity, heteroresistance emergence, and resistance mechanism of omadacycline (OMC) in clinical Enterococcus faecalis isolates from China. A total of 276 isolates were collected retrospectively in China from 2011 to 2015. The MICs of OMC, doxycycline (DOX), and minocycline (MIN) against E. faecalis were determined by broth microdilution. Tetracycline (TET)-specific resistance genes and multilocus sequence typing (MLST) of the isolates were investigated using PCR. The detection frequency of OMC heteroresistance in E. faecalis was evaluated with population analysis profiling (PAP). The mechanism of OMC heteroresistance and resistance in E. faecalis was examined by amplifying 30S ribosomal subunit genes, RNA sequencing (RNA-Seq), and *in vitro* recombination experiments. The OMC MICs of clinical E. faecalis isolates ranged from ≤0.06 to 1.0 mg/liter, and 42% of the E. faecalis isolates with an OMC MIC of 1.0 mg/liter were found to be sequence type 16 (ST16). Six OMC-heteroresistant isolates with MIC values of ≤0.5 mg/liter were detected among 238 E. faecalis isolates. The resistant subpopulations of heteroresistant isolates showed OMC MICs in the range of 2 to 4 mg/liter and were found without 30S ribosomal subunit gene mutations. Moreover, RNA sequencing and *in vitro* recombination experiments demonstrated that overexpression of a bone morphogenetic protein (BMP) family ATP-binding cassette (ABC) transporter substrate-binding protein, OG1RF_RS00630, facilitated OMC heteroresistance in E. faecalis. In conclusion, OMC exhibited better activity against clinical E. faecalis isolates from China than that of DOX or MIN, and overexpression of OG1RF_RS00630 in E. faecalis facilitated the development of OMC heteroresistance.

## Abstract (precis)

This study aimed to evaluate the *in vitro* antimicrobial activity, heteroresistance emergence, and resistance mechanism of omadacycline (OMC) in clinical Enterococcus faecalis isolates from China. A total of 276 isolates were collected retrospectively in China from 2011 to 2015. The MICs of OMC, doxycycline (DOX), and minocycline (MIN) against E. faecalis were determined by broth microdilution. Tetracycline (TET)-specific resistance genes and multilocus sequence typing (MLST) of the isolates were investigated using PCR.

## INTRODUCTION

Enterococcus is a clinically significant pathogen of opportunistic infections, which include urinary tract infections, cholecystitis, endocarditis, bacteremia, and other infections of surgical sites and wounds. Approximately 80% of enterococcal infections are caused by Enterococcus faecalis, and the number of E. faecalis infections appears to be increasing in recent years (1–3). E. faecalis often exhibits resistance to several common antibiotics, such as cephalosporin, aminoglycosides, and sulfamethoxazole, through natural or acquired resistance mechanisms (4, 5). Recently, several reports have shown that the increased incidence of multidrug-resistant enterococci, including vancomycin (VAN)-resistant and linezolid (LZD)-resistant strains, has limited our treatment choices, and controlling multidrug-resistant E. faecalis infections has become a critical need in clinics (4–6).

Omadacycline (OMC) has been recently developed and is a first-in-class aminomethylcycline antibiotic with broad-spectrum activity against Gram-positive and Gram-negative aerobic, anaerobic, and atypical pathogens, including Staphylococcus, Enterococcus, Legionella, and Chlamydia species (7–10). Oral and intravenous OMC formulas have been evaluated in phase III clinical trials and have shown excellent efficacy in the treatment of acute skin and soft tissue infections and community-acquired pneumonia (10–13). OMC differs from earlier tetracycline (TET) derivatives—including doxycycline (DOX), minocycline (MIN), and expanded-spectrum glycylcycline antibiotics such as tigecycline (TGC)—owing to two major structural modifications. Recent reports have demonstrated the *in vitro* active antibacterial potency of OMC with MICs of ≤0.25 mg/liter against various Gram-positive microbes, including enterococci, methicillin-resistant Staphylococcus aureus, and multidrug-resistant Streptococcus pneumoniae (12–14). However, the antimicrobial activity of OMC against clinical isolates of E. faecalis from China has not been established.

Both OMC and TGC represent new derivatives of the TET class of antimicrobial drugs. These drugs are regarded as last-resort antimicrobial treatments for infections by difficult-to-treat bacteria, such as multidrug-resistant E. faecalis. TGC resistance has been linked to genetic mutations that affect the 30S ribosomal subunit of TET binding sites—including mutations that affect the genes encoding 16SrRNA (four copies) and ribosomal protein S10—as well as the overexpression of genes that encode efflux pump regulators, such as *soxS*, *marA*, *ramA*, and *robA* (15–18). Moreover, overexpression of *tet*(M) and *tet*(K) has been shown to increase TGC MIC values and favor the evolution of resistance in Enterococcus faecium (19).

Heteroresistance, which refers to the presence of subpopulations of bacterial cells with higher levels of antibiotic resistance than those of the surrounding populations in the same culture, can lead to the evolution of antibiotic resistance and treatment failure (20). Subpopulation analysis of OMC heteroresistance in E. faecalis can provide information about the risk of antibacterial resistance under antibiotic pressure. The incidence of OMC heteroresistance and its mechanisms in E. faecalis remain unclear. The extent to which TET/TGC resistance mechanisms may contribute to OMC heteroresistance and resistance evolution needs to be determined.

In the present study, we examined the *in vitro* antimicrobial activity of OMC against clinical E. faecalis isolates collected from patients in China. The clonality and OMC susceptibility with respect to the sequence type (ST) were analyzed. Furthermore, population analysis profiling (PAP), molecular sequencing techniques, and *in vitro* functional tests were performed to explore the incidence and underlying mechanism of OMC heteroresistance in clinical E. faecalis isolates. Particular attention was given to mutations that affect the 30S ribosome unit in E. faecalis strains with OMC-induced resistance.

## RESULTS

### *In vitro* antimicrobial activity of OMC against clinical E. faecalis isolates.

The clinical E. faecalis strains were isolated from various infective sample sources, including urine (48.6%), wound secretions (17.0%), blood (11.2%), and bile (7.3%), among others (Fig. S1). Moreover, the OMC MIC data of those isolates were obtained and are summarized in Table 1. Briefly, the data indicated that OMC had robust *in vitro* antimicrobial activity against E. faecalis, and the clinical E. faecalis isolates exhibited a high frequency of resistance to TET, gentamicin (GEN), and erythromycin (ERY). The range of OMC MIC values against E. faecalis was 0.06 to 1.0 mg/liter. We obtained lower MIC values (MIC_50/90_ = 0.5/1.0 mg/liter) for OMC than for DOX and MIN (MIC_50/90_ of 16/32 mg/liter). Notably, 64 LZD-nonsusceptible E. faecalis isolates, including 51 LZD-intermediate and 13 LZD-resistant isolates, and two VAN-intermediate strains were identified. In addition, OMC exhibited excellent antimicrobial activity (MIC ≤ 1 mg/liter) against all LZD- or VAN-nonsusceptible strains (Table 1).

**TABLE 1:** Comparison of *in vitro* antimicrobial activity of OMC and various common antibiotics against E. faecalis

Antibiotic	Resistance rate (%)	MIC breakpoint (μg/ml)	No. of isolates	MIC distribution and statistics for:														
				OMC					DOX					MIN				
				No. of isolates with MIC (mg/liter) of:			Range (mg/liter)	MIC_50/90_ (mg/liter)	No. of isolates with MIC (mg/liter) of:			Range (mg/liter)	MIC_50/90_ (mg/liter)	No. of isolates with MIC (mg/liter) of:			Range (mg/liter)	MIC_50/90_ (mg/liter)
				≤0.25	0.5	≥1			≤4	8	≥16			≤4	8	≥16		
Total (276)				72	166	38	≤0.125–1	0.5/1	39	18	219	0.125–32	16/32	42	30	204	0.125–32	16/32
Tetracycline	83.7	≤4	39	17	21	1	0.125–1	0.5/0.5	35	4	0	0.125–8	0.25/4	37	1	1	0.125–16	0.125/4
		8	6	2	4	0	0.125–0.5	0.5/0.5	2	0	4	4–16	4/16	2	1	3	4–16	8/16
		≥16	231	53	141	37	≤0.125–1	0.5/1	2	14	215	4–32	16/32	3	28	200	0.25–32	16/32
Linezolid	4.7	≤2	212	59	124	29	≤0.125–1	0.5/1	33	16	163	0.125–32	16/32	36	25	151	0.125–32	16/32
		4	51	10	32	9	≤0.125–1	0.5/1	5	1	45	0.125–32	16/32	5	2	44	0.125–32	16/32
		≥8	13	3	10	0	0.125–0.5	0.5/0.5	1	1	11	0.5–32	16/32	1	3	9	0.125–32	16/16
Vancomycin	0	≤4	274	71	165	38	≤0.125–1	0.5/1	38	18	218	0.125–32	16/32	41	30	203	0.125–32	16/32
		8–16	2	1	1	0	0.125–0.5		1	0	1	0.25–16		1	0	1	0.5–16	
Ampicillin	0.4	≤8	275	71	166	38	≤0.125–1	0.5/1	38	18	219	0.125–32	16/32	41	30	204	0.125–32	16/32
		≥16	1	1	0	0	0.125		1	0	0	0.25		1	0	0	0.5	
Gentamycin	68.5	≤4	40	19	18	3	≤0.125–1	0.5/0.5	13	3	24	0.125–32	16/32	12	5	23	0.125–32	16/16
		8	47	14	27	6	≤0.125–1	0.5/1	10	4	33	0.125–32	16/32	12	4	31	0.125–32	16/32
		≥16	189	39	121	29	≤0.125–1	0.5/1	16	11	162	0.125–32	16/32	18	21	150	0.125–32	16/32
Erythromycin	76.8	≤0.5	8	1	6	1	0.25–1	0.5/1	5	1	2	0.125–16	0.25/16	5	1	2	0.125–16	0.125/16
		1–4	56	21	29	6	0.25–1	0.5/1	18	2	36	0.125–32	16/32	20	4	32	0.125–32	16/32
		≥8	212	50	131	31	≤0.125–1	0.5/1	16	15	181	0.125–32	16/32	17	25	170	0.125–32	16/32
Ciprofloxacin	26.1	≤1	168	48	98	22	≤0.125–1	0.5/1	22	14	132	0.125–32	16/32	23	17	128	0.125–32	16/32
		2	36	7	23	6	0.125–1	0.5/1	10	1	25	0.125–32	16/32	10	1	25	0.125–32	16/32
		≥4	72	17	45	10	≤0.125–1	0.5/1	7	3	62	0.125–32	16/32	9	12	51	0.125–32	16/32
Nitrofurantoin	0.7	≤32	270	71	162	37	≤0.125–1	0.5/1	38	17	215	0.125–32	16/32	41	29	200	0.125–32	16/32
		64	4	1	2	1	0.25–1	0.5/1	0	0	4	16–32	16/32	0	0	4	16–32	16/32
		≥128	2	0	2	0	0.5		1	1	0	4–8		1	1	0	4–8	
Trimethoprim-sulfamethoxazole	14.1	≤2/38	237	66	140	31	≤0.125–1	0.5/1	35	16	186	0.125–32	16/32	39	22	176	0.125–32	16/32
		≥4/76	39	6	26	7	0.25–1	0.5/1	4	2	33	0.25–32	16/32	3	8	28	0.125–32	16/32

The distribution of TET-specific resistance genes in clinical E. faecalis isolates is shown in Table 2. The OMC MIC_50/90_ values of 0.5/1 mg/liter were obtained in E. faecalis isolates with at least one of the TET-specific resistance genes, which included *tet*(M), *tet*(K), and *tet*(L), suggesting that the presence of these genes did not affect OMC sensitivity in E. faecalis. In contrast, DOX and MIN MICs in E. faecalis isolates harboring *tet*(M), *tet*(K), or *tet*(L) genes were 16- to 32-fold higher than those of strains without these genes. Four TET-specific resistance genes, namely *tet*(O), *tet*(S), *tet*(W), and *tet*(U), were not found in any E. faecalis isolates (Table 2).

**TABLE 2:** *In vitro* antimicrobial activity of OMC against E. faecalis with TET-specific resistance genes

TET resistance gene(s)	Total no. of isolates	MIC distribution and statistics for:														
		OMC					DOX					MIN				
		No. of isolates with MIC (mg/liter) of:			MIC range (mg/liter)	MIC_50/90_ (mg/liter)	No. of isolates with MIC (mg/liter) of:			MIC range (mg/liter)	MIC_50/90_ (mg/liter)	No. of isolates with MIC (mg/liter) of:			MIC range (mg/liter)	MIC_50/90_ (mg/liter)
		≤0.25	0.5	≥1			≤4	8	≥16			≤4	8	≥16		
*tet*(M)	162	40	104	18	≤0.125–1	0.5/1	5	14	143	0.25–32	16/32	5	23	134	0.5–32	16/32
*tet*(L)	5	2	1	2	0.125–1	0.5/1	2	0	3	0.125–32	16/32	2	0	3	0.125–32	16/32
*tet*(M), *tet*(L)	60	16	31	13	≤0.125–1	0.5/1	2	1	57	4–32	32/32	4	3	53	0.25–32	16/32
*tet*(M), *tet*(K)	4	0	2	2	0.5–1	0.5/1	0	0	4	16	16/16	0	2	2	8–16	8/16
*tet*(M), *tet*(L), *tet*(K)	1	0	0	1	1		0	0	1	32		0	0	1	16	
None detected	44	14	28	2	0.125–1	0.5/0.5	30	3	11	0.125–32	0.5/16	31	3	10	0.125–32	0.25/16

### Clonality of OMC MIC distribution.

A total of 41 STs were identified among the E. faecalis isolates in this study. The predominant STs were ST16 (79/276; 28.6%) and ST179 (71/276; 25.7%) (Table 3 and Fig. S2). With respect to the relationship between ST and the OMC MIC, 20.2% of ST16 strains and only 8.4% of ST179 strains had an OMC MIC level of 1 mg/liter. Conversely, a large proportion (16/38; 42.1%) of the isolates with an OMC MIC level of 1 mg/liter were ST16 strains, demonstrating clonal clustering toward the ST16 genotype (Table 3).

**TABLE 3:** The relationship among OMC, DOX, and MIN MIC distributions with ST16 and ST179 genotypes in E. faecalis

Genotype	No. (%) of isolates	No. (%) of isolates with MIC (mg/liter) for:								
		OMC			DOX			MIN		
		≤0.25	0.5	≥1	≤4	8	≥16	≤4	8	≥16
ST16	79 (28.6)	10 (12.7)	53 (67.1)	16 (20.2)	0	4 (5.1)	75 (94.9)	1 (1.3)	9 (11.4)	69 (87.3)
ST179	71 (25.7)	26 (36.7)	39 (54.9)	6 (8.4)	1 (1.4)	7 (9.9)	63 (88.7)	1 (1.4)	8 (11.3)	62 (87.3)

### OMC heteroresistance.

PAP was performed as a standard method to determine OMC heteroresistance. Overall, six OMC-heteroresistant isolates (EF16C361, EF16C291, EF16C3, EF16C28, EF16C350, and EF16C185) were detected among 238 clinical E. faecalis isolates with MIC values of ≤0.5 mg/liter. Their resistant subpopulations (EF16C361-RS, EF16C291-RS, EF16C3-RS, EF16C28-RS, EF16C350-RS, and EF16C185-RS) were selected from the 1 mg/liter OMC concentrations of the PAP tests. The OMC and TGC MICs of resistant subpopulations were 4- to 8-fold higher than those of the parental isolates, indicating similar elevations of MIC values across both antibiotics (Table 4). The OMC MICs of resistant subpopulations were decreased by 4- to 8-fold after 10 passages on antibiotic-free medium, which suggested that these subpopulations lacked stable mutations conferring OMC resistance and could be reversed to the susceptible phenotype after the removal of antibiotic pressure (Table S1). Moreover, the OMC MICs for these resistant subpopulations could be significantly reduced to ≤0.03 mg/liter and 0.5 mg/liter with the addition of the efflux pump inhibitors (EPIs) carbonyl cyanide *m*-chlorophenylhydrazone (CCCP) and Phe-Arg-β-naphthylamide (PaβN), respectively. This indicates that EPIs could potentiate OMC activity in E. faecalis (Table 4).

**TABLE 4:** Antibiotic susceptibility and resistance mechanisms of the heteroresistant parental isolates, their resistant subpopulations, and strains with OMC-induced resistance

Strain	ST	MIC (mg/liter) for:^*c*^				Mutation(s)^*d*^				
		OMC plus CCCP^*a*^	OMC plus PAβN	OMC	TGC	RR1	RR2	RR3	RR4	S10
EF16C361	179	–	–	0.5	0.25	W	W	W	W	W
EF16C361-RS^*b*^		≤0.03	0.5	4	2	W	W	W	W	W
EF16C361-IR		–	–	8	8	A1024C	T1088G/A1461G	A89G/C1265T/C1191T	W	W
EF16C291	16	–	–	0.5	0.5	W	W	W	W	W
EF16C291-RS		≤0.03	0.5	4	2	W	W	W	W	W
EF16C291-IR1		–	–	8	4	C1261T	G272A/A1461G	C1265T/C1191T	W	W
EF16C291-IR2		–	–	16	4	C988A/C1261T	G272A/G809A	C1265T/C1191T	W	W
EF16C3	16	–	–	0.5	0.5	W	W	W	W	W
EF16C3-RS		≤0.03	0.5	2	2	W	W	W	W	W
EF16C3-IR1		–	–	16	16	W	W/A1461G	C1265T/C1191T	W	W
EF16C3-IR2		–	–	16	16	W	W/A1461G	C1265T/C1191T	W	W
EF16C28	179	–	–	0.5	0.25	W	W	W	W	W
EF16C28-RS		≤0.03	0.5	4	2	W	W	W	W	W
EF16C28-IR		–	–	32	16	W	T1041A/A1461G	C1265T/C1191T	W	W
EF16C350	480	–	–	0.5	0.5	W	W	W	W	W
EF16C350-RS		≤0.03	0.5	4	2	W	W	W	W	W
EF16C350-IR		–	–	16	4	W	G878A	C1265T/C1191T	G811A/A1148G	W
EF16C185 (EF16)	16	–	–	0.5	0.5	W	W	W	W	W
EF16C185-RS (EF16-O2)		≤0.03	0.5	4	2	W	W	W	W	W
EF16C185-IR		–	–	8	4	C1261T/C743T	W	C860T/G1191A/G1265A/C1191T	W	W
OG1RF		–	–	0.125	0.125	W	W	W	W	W
OG1RF-IR1		–	–	32	32	W	A1461G	C1265T/C1191T	C1416T	LYS57ARG
OG1RF-IR2		–	–	32	32	W	C914G/ATGA921-924TGAC/A1461	C1265T/C1191T	W	HIS56TYR, LYS57ARG

a CCCP, carbonyl cyanide *m*-chlorophenylhydrazone; PaβN, Phe-Arg-β-naphthylamide.

b RS, OMC-resistant subpopulations; IR, isolates with OMC-induced resistance.

c –, not determined.

d In individual copies of 16S rRNA (RR1 to RR4) and the 30S ribosomal protein S10. W, wild-type.

### Detection of 30S ribosomal unit mutations in OMC heteroresistant and resistant E. faecalis.

Ten strains with OMC-induced resistance (EF16C361-IR, EF16C291-IR1, EF16C291-IR2, EF16C3-IR1, EF16C3-IR2, EF16C28-IR, EF16C350-IR, EF16C185-IR, OG1RF-IR1, and OG1RF-IR2) were derived from the *in vitro* OMC induction of six heteroresistant parental isolates and the OG1RF strain. The genetic mutations of 30S ribosomal units were then determined in OMC-heteroresistant parental isolates, their resistant subpopulations, and strains with OMC-induced resistance. No genetic mutations of the 30S ribosomal subunits (four 16S rRNA copies and 30S ribosomal protein S10) were found in any of the OMC-heteroresistant parental isolates and their resistant subpopulations. In contrast, mutations in the 16S rRNA genes (C1261T polymorphism in RR1; A1461G polymorphism in RR2; C1265T and C1191T polymorphisms in RR3) were detected in strains with OMC-induced resistance. Furthermore, 30S ribosomal protein S10 mutations were found in only two strains with OMC-induced resistance (OG1RF-IR1 and OG1RF-IR2) (Table 4).

### Differentially expressed genes between the heteroresistant parental isolate and its resistant subpopulation.

**FIG 1:**
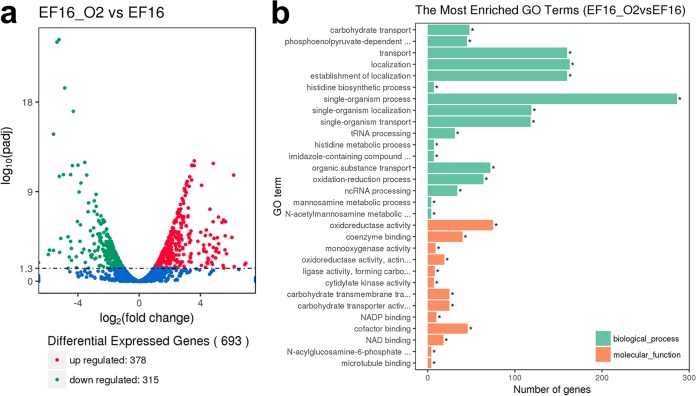
Differentially expressed genes (DEGs) between heteroresistant parental strain EF16 and its resistant subpopulation EF16-O2. (a) Distribution of DEGs. Red, green, and blue colors represent upregulated and downregulated genes and those genes not differentially expressed, respectively. The *x* axis represents fold change and the *y* axis represents the *P* value of DEGs. (b) Functional analysis of DEGs between the EF16-O2 and EF16 strains.

Because the heteroresistance mechanism in this study was elusive, we performed RNA sequencing (RNA-Seq) to compare the transcription of unique genes between the heteroresistant parental strain EF16C185 (EF16) and its resistant subpopulation EF16C185-RS (EF16-O2). Before RNA sequencing, the growth curves of EF16 and EF16-O2 were determined, and the growth rates of these two strains showed no significant difference (Fig. S3). A total of 693 differentially expressed genes (DEGs), including 378 upregulated and 315 downregulated genes, were identified in EF16-O2 compared to EF16 in RNA sequencing analysis (Fig. 1a and Tables S2 and S3). Mapping of these DEGs using gene ontology (GO) analysis showed that the most frequent pathways were related to carbohydrate metabolism (31 genes), cationic antimicrobial peptide resistance (21 genes), fructose/mannose metabolism (31 genes), the phosphotransferase system (38 genes), and terpenoid backbone biosynthesis (9 genes) (Fig. 1b).

**FIG 2:**
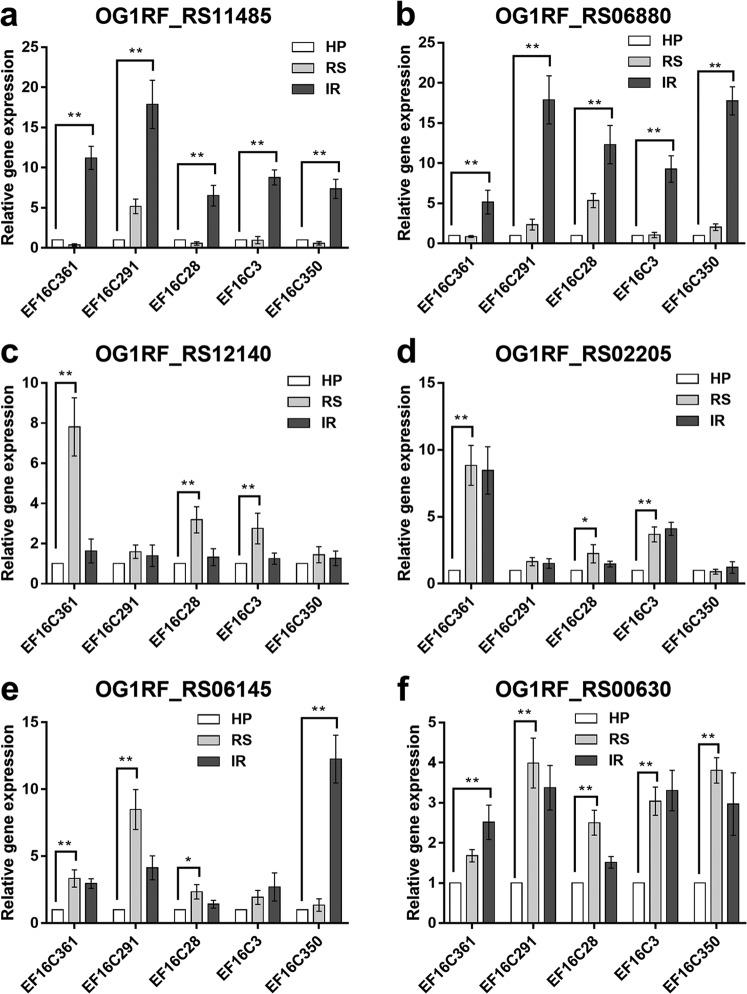
Relative transcriptional analysis of differentially expressed genes (DEGs) used in overexpression experiments. Relative expressions of OG1RF_RS11485 (a), OG1RF_RS06880 (b), OG1RF_RS12140 (c), OG1RF_RS02205 (d), OG1RF_RS06145 (e), and OG1RF_RS00630 (f) were determined by qRT-PCR analysis. The housekeeping gene *recA* was used as the endogenous reference gene. The heteroresistant parental strain was used as the reference strain (expression = 1.0). All qRT-PCRs were carried out in triplicate with three independent RNA samples. HP, OMC-heteroresistant parental strains; RS, OMC-resistant subpopulations; IR, strains with OMC-induced resistance. **, *P < *0.01; *, *P < *0.05.

The OMC MICs in resistant subpopulations of heteroresistant isolates could be reversed to sensitive levels by adding an EPI, supporting the notion that membrane proteins or efflux pumps may participate in the initial upregulation of OMC MIC values during the progression of heteroresistance. Twelve upregulated DEGs, which showed potential associations with heteroresistance, were validated by quantitative real-time (qRT-PCR) in EF16 and EF16-O2 (Table 5). To further validate their impact on OMC susceptibility, the expression levels of those 12 DEGs were investigated in the remaining five heteroresistant parental isolates, their resistant subpopulations, and strains with OMC-induced resistance (Fig. 2 and Fig. S4). Overexpression of six candidate genes, namely, OG1RF_RS00630, OG1RF_RS12140, OG1RF_RS02205, OG1RF_RS06145, OG1RF_RS06880, and OG1RF_RS11485, was correlated with the occurrence of OMC heteroresistance or resistance (Fig. 2).

**TABLE 5:** Transcriptional expression levels of 12 candidate differentially expressed genes between EF16-O2 and EF16^*b*^

Gene identifier	Description or predicted function	Expression ratio (EF16-O2/ EF16) according to:	
		RNA-Seq (*P* value)^*a*^	qRT-PCR^*c*^
OG1RF_RS11300	ABC transporter substrate-binding protein	2.48 (0.0013)	1.28 ± 0.17
OG1RF_RS11485	Ribose transporter RbsU	2.62 (0.0006)	1.40 ± 0.11
OG1RF_RS10660	ABC-F type ribosomal protection protein Lsa(A)	2.77 (0.011)	2.16 ± 0.14
OG1RF_RS06880	CoA-binding protein	3.37 (0.0003)	2.67 ± 0.35
OG1RF_RS12140	ABC transporter ATP-binding protein	3.75 (<0.0001)	15.70 ± 2.48
OG1RF_RS02205	ABC transporter ATP-binding protein	3.84 (<0.0001)	3.22 ± 0.49
OG1RF_RS05865	Sulfate ABC transporter ATP-binding protein	4.21 (<0.0001)	8.54 ± 1.24
OG1RF_RS06145	Molybdate ABC transporter substrate-binding protein	5.25 (<0.0001)	21.47 ± 4.53
OG1RF_RS09080	Sugar ABC transporter substrate-binding protein	5.40 (<0.0001)	39.61 ± 5.46
OG1RF_RS12590	ABC transporter permease	6.60 (<0.0001)	6.41 ± 0.79
OG1RF_RS00350	Alkaline shock response membrane anchor protein AmaP	6.72 (<0.0001)	2.23 ± 0.21
OG1RF_RS00630	BMP family ABC transporter substrate-binding protein	7.73 (<0.0001)	4.49 ± 0.64

a The DEGs of RNA sequencing were defined by a change ratio of ≥2 and a *P* value of <0.05.

b According to RNA sequencing and qRT-PCR.

c qRT-PCR data are given as means ± standard deviations of results from three independent experiments.

### Overexpression of OG1RF_RS00630 in E. faecalis contributes to the occurrence of OMC heteroresistance.

To confirm the roles of the candidate genes (OG1RF_RS12140, OG1RF_RS02205, OG1RF_RS06145, OG1RF_RS11485, OG1RF_RS06880, and OG1RF_RS00630) in OMC heteroresistance and resistance in E. faecalis, the overexpression of these genes in OMC-sensitive isolates was conducted. The overexpression plasmids pRS12140, pRS02205, pRS06145, pRS11485, pRS06880, and pRS00630 were constructed and transformed into OMC-sensitive isolates (three per gene), showing low expression level of the target gene (Table S4 and Table S5). Stable overexpression of the candidate genes in the transformed strains was confirmed by qRT-PCR (Fig. S5). Overexpression of the six candidate genes did not induce OMC MIC elevation in the absence of antibiotic pressure. However, PAP showed that overexpression of OG1RF_RS00630 led to the occurrence of the OMC-heteroresistant phenotype in all three OMC-sensitive E. faecalis isolates, compared with negative findings in the parental isolate controls (Table 6). Phylogenetic and homology analyses showed that E. faecalis OG1RF_RS00630 encodes a bone morphogenetic protein (BMP) family ATP-binding cassette (ABC) transporter substrate-binding protein that is ubiquitous in a variety of bacterial species and shares high identity (over 60%) with those of other Gram-positive bacteria (Fig. S6 and Table S6). Our data indicate that the product of this gene may contribute to OMC heteroresistance in E. faecalis.

**TABLE 6:** OMC MICs in E. faecalis derivatives with overexpressing candidate genes and their influence on population analysis profiling

Transformed plasmid	Isolate	OMC MIC (mg/liter) for:			PAP test results for:		
		Parental strain^*a*^	Vector control strain^*b*^	Derivative strain^*c*^	Parental strain	Vector control strain	Derivative strain
pRS00630	EF16C2	0.5	0.5	0.5	Negative	Negative	Positive
	EF16C105	0.5	0.5	0.5	Negative	Negative	Positive
	EF16C284	0.25	0.25	0.125	Negative	Negative	Positive
pRS12140	EF16C105	0.5	0.5	0.5	Negative	Negative	Positive
	EF16C39	0.5	0.25	0.25	Negative	Negative	Negative
	EF16C40	0.25	0.25	0.25	Negative	Negative	Negative
pRS02205	EF16C2	0.5	0.5	0.5	Negative	Negative	Negative
	EF16C105	0.5	0.5	0.5	Negative	Negative	Negative
	EF16C39	0.5	0.25	0.5	Negative	Negative	Negative
pRS06145	EF16C2	0.5	0.5	0.5	Negative	Negative	Negative
	EF16C105	0.5	0.5	0.5	Negative	Negative	Negative
	EF16C283	0.5	0.5	0.25	Negative	Negative	Positive
pRS06880	EF16C2	0.5	0.5	0.5	Negative	Negative	Negative
	EF16C105	0.5	0.5	0.5	Negative	Negative	Negative
	EF16C284	0.25	0.25	0.25	Negative	Negative	Negative
pRS11485	EF16C2	0.5	0.25	0.25	Negative	Negative	Negative
	EF16C105	0.5	0.5	0.5	Negative	Negative	Negative
	EF16C39	0.5	0.5	0.25	Negative	Negative	Negative

a The parental strain was an OMC-sensitive E. faecalis isolate without an OMC-heteroresistant phenotype according to the PAP test.

b The vector control strain was the parental strain transformed with the pIB166 vector.

c The derivative strain was the parental strain transformed with each overexpression plasmid.

## DISCUSSION

In the present study, OMC exhibited excellent *in vitro* antimicrobial activity against LZD-nonsusceptible E. faecalis, indicating potential application of OMC in the treatment of multidrug-resistant E. faecalis infections. E. faecalis maintained low OMC MICs in TET-resistant strains even as DOX and MIN MICs increased. The present findings support the efficacy of OMC against clinically infective E. faecalis in China (17, 18). The MIC_50/90_ of OMC in 276 E. faecalis isolates was 0.5/1.0 mg/liter in the present study, which was higher than that reported in previous studies (≤0.125/0.125 mg/liter) (7, 9, 13). This difference suggests that susceptibility of E. faecalis to OMC varies across geographical regions, which may lead to regional differences in clinical perspectives. The main STs observed in our sample were ST16 and ST179, which is consistent with prior reports (21, 22). Our data indicate the clonality of E. faecalis, with clustering of the 1 mg/liter OMC MIC level in the ST16 genotype. This underscores the risk of nosocomial transmission of E. faecalis strains with high OMC MICs.

The most common TET resistance mechanisms in Gram-positive and Gram-negative pathogens include ribosomal protection and efflux pump proteins. Notably, the dissemination of these TET-specific resistance factors has limited the clinical utility of TET and its derivatives, including DOX and MIN (17, 18). Antimicrobial susceptibility of new TET drugs, such as TGC and eravacycline, might be slightly influenced by these two mechanisms. The overexpression of some TET-specific resistance genes, including the genes that encode ribosomal protection and efflux pump proteins, have been reported to reduce TGC susceptibility (19). Our data showed that the ribosomal protection protein gene, *tet*(M), and the efflux pump genes, *tet*(K) and *tet*(L), did not affect OMC susceptibility. This finding indicates that OMC might have overcome these two common resistance mechanisms.

Although OMC retained low MIC values against clinical E. faecalis isolates in this study, monitoring the incidence of heteroresistance can provide information about the potential risk of OMC resistance in the future (23, 24). Six OMC-heteroresistant isolates were detected among 238 clinical E. faecalis isolates with MIC values of ≤0.5 mg/liter according to PAPs. The OMC and TGC MIC values were elevated synchronously in OMC-resistant subpopulations, pointing to a cross-resistance risk for these two antibiotics. Therefore, recognition of the development of OMC resistance in E. faecalis may be facilitated by monitoring OMC heteroresistance in clinical isolates. Furthermore, the OMC MICs of resistant subpopulations could be reversed after 10 passages on antibiotic-free medium, while those of isolates with OMC-induced resistance remained stable. This indicates that the resistant subpopulations were reversible and not the result of a stable mutation, which is consistent with the findings of previous research (25).

X-ray crystallography of TET in complex with the 30S ribosomal subunit of Thermus thermophilus revealed two TET binding sites on the 30S subunit (26, 27). As TGC and OMC are new TET derivatives, they are thought to target similar binding sites as TET. However, competition studies with radiolabeled TET indicate that OMC can inhibit *in vitro* translation at half the concentration required for TET (17), suggesting that OMC may have a 2-fold stronger affinity than TET for the ribosome. Mutations affecting TET binding sites of the 30S ribosomal subunit have been shown to confer TET and TGC resistance in several bacterial species (15–17). The mutational characteristics of OMC-induced resistance in E. faecalis in this study were consistent with the prior identification of various target-interaction related mutations in individual copies of 16S rRNA in bacteria with TGC-induced resistance (16). We detected mutations in all four 16S rRNA gene copies in E. faecalis under OMC pressure. Furthermore, strains having a greater number of genetic mutations in 16S rRNA copies tended to have greater OMC and TGC resistance. Mutations in the gene encoding the 30S ribosomal protein S10 occurred at a relatively low frequency (2/10; 20%) in our strains with OMC-induced resistance, compared to prior findings in multiple bacterial species under TGC pressure (15), indicating that the role of S10 in the evolution of OMC resistance needs to be further studied.

The lack of genetic mutations in 30S ribosomal subunits of OMC-heteroresistant parental isolates and their resistant subpopulations indicates that the MIC elevation observed with the emergence of OMC heteroresistance cannot be explained by such mutations. The TGC resistance and heteroresistance in several Gram-negative bacterial species have been linked to the overexpression of several efflux pump proteins (e.g., SoxS, MarA, RamA, and RobA) (28, 29). Our data showed that the addition of an EPI could restore sensitivity to OMC in resistant subpopulations of OMC-heteroresistant isolates, indicating that efflux pumps may be involved in OMC heteroresistance under antibiotic pressure. Following both RNA sequencing and qRT-PCR analyses, 12 upregulated DEGs encoding efflux pump proteins or membrane proteins were observed in the resistant subpopulations compared with the parental strains. Furthermore, *in vitro* recombination experiments indicated that overexpression of the candidate gene OG1RF_RS00630 favored OMC heteroresistance in E. faecalis but did not affect OMC and TGC MICs in the absence of antibiotic pressure. Phylogenetic and homology analyses indicated that the efflux protein encoded by OG1RF_RS00630 is an ATP-binding cassette family protein expressed mainly in the cellular membrane. A previous study showed that the ortholog of OG1RF_RS00630 (EF0177) from E. faecalis V583 is involved in ABC transporter-mediated ribonucleoside uptake (30). Our data demonstrate for the first time that OG1RF_RS00630 participates in the development of OMC heteroresistance in E. faecalis.

In conclusion, OMC exhibited robust antimicrobial effects against clinical E. faecalis isolates from China, with lower MIC_50/90_ values than those of DOX or MIN. Furthermore, OMC showed excellent *in vitro* antimicrobial activity to LZD-nonsusceptible clinical E. faecalis isolates. Isolates with OMC MICs of 1 mg/liter showed ST16 clonality. Mutations in 30S ribosome units were associated with OMC resistance, and overexpression of a BMP family ABC transporter substrate-binding protein (OG1RF_RS00630) in E. faecalis appeared to favor the occurrence of OMC heteroresistance. Moreover, the emergence of heteroresistance among clinical E. faecalis isolates in China, especially those with high OMC MICs, is noteworthy as a possible harbinger of future resistance development.

## MATERIALS AND METHODS

### Bacterial isolates, growth conditions, and chemicals.

A total of 276 nonduplicate clinical E. faecalis strains were collected retrospectively from inpatients at Shenzhen Nanshan People’s Hospital (a tertiary-care teaching center hospital in China with 1,200 beds) from 1 January 2011 to 31 December 2015. Bacterial species were identified by standard methods using a Vitek 2 compact system (bioMérieux, Marcy l’Etoile, France). E. faecalis ATCC 29212 was used as the quality control strain. All procedures performed were approved by the ethical standards of Shenzhen Nanshan People’s Hospital. The E. faecalis strains were cultured in tryptic soy broth (TSB; Oxoid, Basingstoke, UK) at 37°C with shaking at 220 rpm. The aminomethylcycline antibiotic OMC was purchased from MedChem Express (Princeton, NJ). DOX, MIN, and TGC were purchased from Aladdin (Shanghai, China).

### Antibiotic susceptibility testing.

Antimicrobial susceptibility of several common antibiotics, including ciprofloxacin (CIP), ampicillin (AMP), nitrofurantoin (NIT), trimethoprim-sulfamethoxazole (SXT), GEN, ERY, LZD, VAN, and TET, were determined by broth microdilution using the Vitek 2 compact system (bioMérieux, Marcy l’Etoile, France). The MICs of OMC, DOX, MIN, and TGC were determined by the broth microdilution method according to Clinical and Laboratory Standards Institute (CLSI) guidelines (CLSI-M100-S26). As CLSI E. faecalis MIC breakpoints for OMC have not yet been established, we employed three MIC levels for OMC, ≤0.25 mg/liter (susceptible), 0.5 mg/liter (intermediate), and ≥1 mg/liter (resistant), based on the FDA recommendations (https://www.fda.gov/drugs/development-resources/omadacycline-injection-and-oral-products).

### Population analysis profiles.

Population analysis profiling (PAP) was used as a reference method to investigate OMC heteroresistance among 238 clinical E. faecalis isolates with OMC MIC values of ≤0.5 mg/liter as described previously (23). Briefly, 50-μl aliquots of cell suspension (corresponding to a 0.5 McFarland standard for *Enterococcus* cultures grown on blood agar plates for 24 h at 37°C; approximately 1 × 10^8^ CFU/ml) were spread onto Mueller-Hinton agar plates, with or without various concentrations of OMC (0.5, 1, 2, 3, 4, and 5 mg/liter). Plates were then incubated at 37°C and colonies were counted after 24 h. According to the FDA breakpoint of OMC MIC values for E. faecalis, OMC heteroresistance was defined as an OMC-susceptible isolate (MIC ≤0.5 mg/liter) with subpopulations growing in the presence of ≥1 mg/liter OMC, with a detection limit of 20 CFU/ml. Three colonies, categorized as the resistant subpopulations of each OMC-heteroresistant isolate, were selected from the 1 mg/liter OMC concentration of the PAP test, and the OMC MICs were reassessed after serial passaging on antibiotic-free medium to evaluate the stability of the heteroresistant phenotype. The OMC-resistant subpopulations or their parental strains were cultured in Mueller-Hinton broth (MHB) either supplemented with 1 mg/liter OMC or without OMC, respectively, for the subsequent experiments.

### *In vitro* induction of OMC-resistance in E. faecalis under OMC pressure.

Six OMC-heteroresistant parental isolates and the OG1RF strain were used to induce OMC-resistant isolates. These isolates were subcultured serially in MHB containing gradually increasing concentrations of OMC, with the initial concentration being MIC values followed by successive increases to 2×, 4×, 8×, and 16× MIC (31). Strains were cultured for four passages before their entry into the next concentration. Isolates from the passages of each concentration were stored at −80°C in MHB containing 40% glycerol, until further determination of genetic mutations and subsequent MIC assays.

### PCR for multilocus sequence typing and detection of TET resistance and 30S ribosomal subunit genes.

Total DNA samples were extracted from E. faecalis isolates in lysis buffer for microorganisms and submitted to direct PCR using the PCR mastermix (Thermo Fisher Scientific, Waltham, MA) according to the manufacturer’s instructions. The multilocus sequence typing (MLST) was determined by PCR and sequence alignment as previously described (21, 32). We also used PCR to detect TET-specific resistance genes, including *tet*(K) and *tet*(L), which encode efflux pump proteins; *tet*(M), *tet*(S), *tet*(O), and *tet*(W), which encode ribosomal protection proteins; and *tet*(U), a putative TET resistance determinant, as previously described (22). Genetic mutations in 30S ribosomal subunits, including four 16S rRNA gene copies and the 30S ribosomal protein S10, were detected by PCR amplification and sequence alignment. The primers used in this study are listed in Tables S7 to S9.

### Efflux pump inhibition.

The role of the efflux pump in the development of OMC heteroresistance was evaluated using the EPIs Phe-Arg-β-naphthylamide (PaβN; Sigma, St. Louis, MO) and carbonyl cyanide *m*-chlorophenylhydrazone (CCCP; Sigma). The OMC MICs of the resistant subpopulations of heteroresistant isolates were determined by agar dilution in the presence and absence of PAβN (50 mg/liter) or CCCP (50 mg/liter), as previously described (28). Efflux pump inhibition of antimicrobial susceptibility was considered significant if the magnitude of the MIC value was decreased at least 4-fold in the presence of EPIs, which is consistent with the methods of previous investigations (24).

### Measurement of bacterial growth curves.

Overnight bacterial cultures of EF16C185 (EF16) or EF16C185-RS (EF16-O2) were diluted 1:200 into 1 ml of fresh MHB or into MHB containing 1 mg/liter OMC, respectively, of which 300 μl was added into each well of a 96-well plate. Three parallel wells were used for each sample. The plates were placed in a Bioscreen C MBR (Oy Growth Curves Ab Ltd., Helsinki, Finland), and the bacteria were grown at 37°C with shaking at 220 rpm. Growth curves of the strains were determined by measuring the optical density at 600 nm (OD_600_) at 30-min intervals over a period of 16 h.

### RNA sequencing.

The heteroresistant parental strain EF16 or its resistant subpopulation EF16-O2 was grown overnight in 20 ml of antibiotic-free MHB or MHB containing 1 mg/liter OMC, respectively. The overnight cultures were diluted 1:100 into 50 ml of MHB under the same conditions and grown to the mid-log phase (4 h) at 37°C. The bacteria were harvested by centrifugation, and total RNA of EF16 and EF16-O2 was isolated using an RNeasy minikit (Qiagen, Hilden, Germany) according to the manufacturer’s instructions (three biological replicates for each strain). The RNA quality and quantity were determined by 1.0% formaldehyde denaturing agarose gel electrophoresis and spectrophotometry in a NanoDrop ND-1000 machine, respectively. RNA sequencing was performed as previously described (33). Briefly, rRNA was removed from total RNA with a Ribo-Zero rRNA removal kit for Gram-positive bacteria (Illumina, San Diego, CA). Fragmented RNA was used as a template for PCR with random primers. To build the cDNA libraries, cDNA fragments were purified using an AMPure XP system (Beckman Coulter, Beverly, USA) to select fragments of ∼150 to 200 bp in length. The PCR was then performed using Phusion high-fidelity DNA polymerase (Thermo Fisher Scientific, Waltham, MA), and the library quality was assessed on an Agilent Bioanalyzer 2100 system (Agilent Technologies, CA). The library preparations were then sequenced on an Illumina HiSeq X Ten platform, and reads of paired-end 150 bp (PE150) were generated. Clean data were obtained by removing rRNA reads, sequencing adapters, short fragment reads, and other low-quality reads from the raw data. The remaining reads were mapped to the reference genome of OG1RF on the NCBI website with Bowtie 2 software. When the reads were aligned, one mismatch with the reference sequence was allowed. The alignments were further processed using BEDTools software to determine the transcript expression levels and their differential expression between each two of the three samples. Differential expression of all transcripts was quantified using DEGseq software, and the fold change values were recorded. Genes with adjusted *P* values (Benjamini-Hochberg method) of <0.05 and at least a 2-fold difference in expression were considered DEGs.

### Quantitative real-time PCR analysis.

Transcriptional levels of OMC heteroresistant candidate genes were determined by qRT-PCR with the primers listed in Table S10, according to previously described methods (24). Briefly, overnight cultures of the bacterial strains were diluted 1:100 into 10 ml of MHB (cultures of the resistant subpopulations supplemented with 1 mg/liter OMC) and grown for 4 h at 37°C. Total bacterial RNA was extracted using an RNeasy minikit (Qiagen, Hilden, Germany), and the extracted RNA was reverse transcribed into cDNA using a PrimeScript real-time (RT) reagent kit (TaKaRa Bio, Inc., Shiga, Japan). qRT-PCR was performed with the SYBR Premix *Ex Taq* II kit (TaKaRa Bio, Inc., Shiga, Japan) in a Mastercycler ep realplex system (Eppendorf, Hamburg, Germany). The internal control gene *recA* was used to normalize the expression of each candidate gene. The threshold cycle (*C_T_*) numbers were determined by the detection system software, and the data were analyzed based on the 2^−ΔΔCT^ method. Expression levels of the target genes were compared with those of the E. faecalis OG1RF strain or the heteroresistant parental strain (expression = 1). All qRT-PCRs were carried out in triplicate with three independent RNA samples.

### Gene overexpression.

Full-length candidate genes, which included OG1RF_RS12140, OG1RF_RS02205, OG1RF_RS06145, OG1RF_RS11485, OG1RF_RS06880, and OG1RF_RS00630, were amplified from the total DNA extracted from OG1RF isolates and then integrated separately into the pIB166 vector (the inserted gene was controlled by a P23 promoter for overexpression) (34). The positive clones were screened by chloramphenicol and verified by PCR and sequencing. The overexpression plasmids (pRS12140, pRS02205, pRS06145, pRS11485, pRS06880, and pRS00630) were then transformed separately into three OMC-sensitive isolates (Table S5) and further identified by PCR and sequencing. The pIB166 vector was transferred into the same isolates as a control. Subsequently, overnight cultures of the derivative and parental strains were diluted 1:100 into antibiotic-free MHB and grown for 4 h at 37°C, and transcriptional levels of candidate genes were measured by qRT-PCR as described above. The OMC MIC values for these derivatives were determined, and heteroresistance was evaluated by PAP determination under OMC pressure as described above. The primers for plasmid construction are listed in Table S11.

### Statistical analysis.

Continuous data were analyzed with the Student’s *t* test and one-way factorial analysis of variance (ANOVAs) in the SPSS software package (version 17.0; Chicago, IL). *P* values of <0.05 were regarded as statistically significant.

### Data availability.

The RNA sequencing data were deposited in the NCBI database under BioProject no. PRJNA505112 and BioSample no. SAMN10411100, SAMN10411101, SAMN10411102, SAMN10411103, SAMN10411104, and SAMN10411105.

## Supplementary Material

Supplemental file 1
