# Supplementary material for: Omadacycline Efficacy against Enterococcus faecalis Isolated in China: In Vitro Activity, Heteroresistance, and Resistance Mechanisms
Source: Antimicrob Agents Chemother. 2020 Feb 21;64(3):e02097-19. doi: 10.1128/AAC.02097-19 (PMC7038293; doi:10.1128/AAC.02097-19)
Supplement: Supplemental file 1 [file AAC.02097-19-s0001.pdf]

Supplementary Electronic Materials

Table S1. The OMC and TGC MICs of the resistant subpopulations and OMC-induced resistant isolates after 10 passages on antibiotic-free medium.

Isolate	MIC (mg/L)					
	OMC			TGC		
	Parental	5 passages	10 passages	Parental	5 passages	10 passages
**OMC resistant subpopulations of heteroresistant isolates**						
EF16C361-RS[a]	4	2	0.5	2	1	0.5
EF16C291-RS	4	2	1	2	1	0.5
EF16C3-RS	2	1	0.5	2	1	0.25
EF16C28-RS	4	2	1	2	0.5	0.25
EF16C350-RS	4	2	1	2	1	0.5
EF16C185-RS	4	1	0.5	2	1	0.5
**OMC-induced resistant isolates**						
EF16C361-IR	8	8	8	8	8	8
EF16C291-IR2	16	16	16	4	8	8
EF16C3-IR2	16	16	16	16	16	16
EF16C28-IR	32	32	32	16	16	16
EF16C350-IR	16	16	8	4	8	8
EF16C185-IR	8	8	8	4	4	4

[a]RS: OMC resistant subpopulations; IR: OMC-induced resistant isolates.

Table S2. Differentially expressed genes (DEGs) of EF16-O2 compared to EF16 by RNA-sequencing at middle-log phase.

Gene id	RNA-seq fold change[a] (EF16-O2/ EF16)	*p*-value[b]	Description or predicted function
OG1RF_RS07070	124.67	0.0032	2-oxo acid dehydrogenase subunit E2
OG1RF_RS07080	119.88	0.0048	thiamine pyrophosphate-dependent dehydrogenase E1 component subunit alpha
OG1RF_RS03915	74.12	0.0085	pyruvate phosphate dikinase
OG1RF_RS02315	72.01	<0.0001	tagatose 1, 6-diphosphate aldolase
OG1RF_RS08270	66.56	<0.0001	glucosamine--fructose-6-phosphate aminotransferase
OG1RF_RS08610	52.24	0.0002	metal ABC transporter substrate-binding protein
OG1RF_RS02310	50.00	<0.0001	PTS fructose transporter subunit IIA
OG1RF_RS08275	49.16	<0.0001	SIS domain-containing protein
OG1RF_RS07770	48.69	0.0001	PTS system mannose/fructose/N-acetylgalactosamine-transporter subunit IIB
OG1RF_RS07085	46.51	0.0128	dihydrolipoyl dehydrogenase
OG1RF_RS04895	45.76	<0.0001	glycine/betaine/sarcosine/D-proline reductase family selenoprotein B
OG1RF_RS07065	44.69	0.0059	PTS sugar transporter subunit IIC
OG1RF_RS07755	42.62	<0.0001	PTS fructose transporter subunit IIA
OG1RF_RS13115	36.91	0.0002	oxaloacetate decarboxylase
OG1RF_RS07095	36.64	<0.0001	branched-chain phosphotransacylase
OG1RF_RS02305	36.31	<0.0001	PTS fructose transporter subunit IIC
OG1RF_RS08280	34.39	<0.0001	PTS mannose transporter subunit IID
OG1RF_RS07765	33.34	<0.0001	PTS sugar transporter subunit IIC
OG1RF_RS13110	32.22	0.0001	NADP-dependent malic enzyme
OG1RF_RS04885	31.93	0.0007	PTS sugar transporter subunit IIB
OG1RF_RS07760	29.58	<0.0001	PTS fructose transporter subunit IID
OG1RF_RS07785	28.78	<0.0001	tagatose-bisphosphate aldolase
OG1RF_RS07800	28.71	<0.0001	glycosyltransferase family 8 protein
OG1RF_RS12075	28.49	<0.0001	hypothetical protein
OG1RF_RS13105	28.14	0.0005	triphosphoribosyl-dephospho-CoA synthase CitG
OG1RF_RS07090	27.59	0.0044	butyrate kinase
OG1RF_RS13125	26.37	0.0023	citrate lyase subunit alpha
OG1RF_RS08290	25.33	<0.0001	PTS mannose/fructose/sorbose transporter subunit IIB
OG1RF_RS07805	24.66	<0.0001	glycosyltransferase family 8 protein
OG1RF_RS08285	24.06	<0.0001	PTS sugar transporter subunit IIC
OG1RF_RS07790	22.83	0.0012	tagatose-6-phosphate ketose
OG1RF_RS13140	22.63	0.0008	[citrate (pro-3S)-lyase] ligase
OG1RF_RS13130	22.29	0.0035	citrate (pro-3S)-lyase subunit beta
OG1RF_RS13120	21.53	0.0027	citrate lyase holo-[acyl-carrier protein] synthase
OG1RF_RS07750	20.97	<0.0001	hypothetical protein
OG1RF_RS08605	20.92	0.0001	metal ABC transporter permease
OG1RF_RS13150	20.18	0.0101	sodium ion-translocating decarboxylase subunit beta
OG1RF_RS04890	19.98	<0.0001	PTS system%2C cellobiose-specific IIC component
OG1RF_RS08265	18.76	0.0001	DUF2200 domain-containing protein
OG1RF_RS07780	18.73	0.0001	tagatose-6-phosphate kinase
OG1RF_RS07775	18.71	0.0001	beta-galactosidase
OG1RF_RS01400	18.51	<0.0001	chitin-binding protein
OG1RF_RS12000	16.87	<0.0001	biotin transporter BioY
OG1RF_RS04880	16.72	0.0060	N(4)-(beta-N-acetylglucosaminyl)-L-asparaginase
OG1RF_RS08295	16.25	<0.0001	PTS fructose transporter subunit IIA

OG1RF_RS01395	14.93	0.0002	chitinase
OG1RF_RS05095	14.27	<0.0001	NADP-dependent malic enzyme
OG1RF_RS00535	12.16	<0.0001	glycosyl hydrolase
OG1RF_RS11090	12.07	<0.0001	chitinase
OG1RF_RS10900	10.89	<0.0001	cobalt ABC transporter permease
OG1RF_RS12585	10.87	<0.0001	membrane protein
OG1RF_RS10015	10.78	<0.0001	YgeY family selenium metabolism-linked hydrolase
OG1RF_RS13580	10.57	0.0023	hypothetical protein
OG1RF_RS06825	10.52	0.0002	hypothetical protein
OG1RF_RS00365	10.48	<0.0001	Asp23/Gls24 family envelope stress response protein
OG1RF_RS04575	10.28	<0.0001	DUF378 domain-containing protein
OG1RF_RS02300	10.15	0.0007	1-phosphofructokinase
OG1RF_RS00990	10.13	0.0005	aldehyde dehydrogenase
OG1RF_RS10935	9.94	<0.0001	hydroxyethylthiazole kinase
OG1RF_RS00370	9.89	<0.0001	GlsB/YeaQ/YmgE family stress response membrane protein
OG1RF_RS00515	9.88	<0.0001	YfcC family protein
OG1RF_RS07735	9.83	<0.0001	hypothetical protein
OG1RF_RS12005	9.83	<0.0001	signal peptidase I
OG1RF_RS10010	9.64	<0.0001	knotted carbamoyltransferase YgeW
OG1RF_RS05100	9.61	0.0020	L-malate permease
OG1RF_RS03925	9.54	<0.0001	phosphotransferase
OG1RF_RS00500	9.27	<0.0001	ornithine carbamoyltransferase
OG1RF_RS01715	9.16	<0.0001	gluconate 5-dehydrogenase
OG1RF_RS00355	9.08	<0.0001	DUF2273 domain-containing protein
OG1RF_RS05935	8.83	<0.0001	dihydrolipoyl dehydrogenase
OG1RF_RS10925	8.60	<0.0001	bifunctional hydroxymethylpyrimidine kinase/phosphomethylpyrimidine kinase
OG1RF_RS05925	8.60	<0.0001	alpha-ketoacid dehydrogenase subunit beta
OG1RF_RS02395	8.49	<0.0001	WxL domain-containing protein
OG1RF_RS12125	8.46	<0.0001	oligopeptide ABC transporter substrate-binding protein
OG1RF_RS08600	8.44	<0.0001	metal ABC transporter ATP-binding protein
OG1RF_RS12295	8.37	<0.0001	PTS mannose/fructose/sorbose transporter subunit IIB
OG1RF_RS13065	8.17	<0.0001	PTS sorbitol transporter subunit IIC
OG1RF_RS09925	8.04	<0.0001	pyruvate:ferredoxin (flavodoxin) oxidoreductase
OG1RF_RS10885	7.93	<0.0001	thiaminase II
OG1RF_RS05115	7.92	<0.0001	NADH peroxidase
OG1RF_RS00630	7.73	<0.0001	BMP family ABC transporter substrate-binding protein
OG1RF_RS05250	7.68	0.0079	beta-glucosidase
OG1RF_RS10005	7.57	<0.0001	carbamate kinase
OG1RF_RS09120	7.56	<0.0001	myo-inosose-2 dehydratase
OG1RF_RS10000	7.52	0.0003	RidA family protein
OG1RF_RS02130	7.51	<0.0001	hypothetical protein
OG1RF_RS00360	7.35	<0.0001	Asp23/Gls24 family envelope stress response protein
OG1RF_RS03960	7.34	<0.0001	6-aminohexanoate hydrolase
OG1RF_RS02190	7.23	<0.0001	type II-A CRISPR-associated protein Csn2
OG1RF_RS04780	7.21	0.0001	aldo/keto reductase
OG1RF_RS05255	7.20	<0.0001	hypothetical protein
OG1RF_RS12290	7.07	0.0021	PTS fructose transporter subunit IIA
OG1RF_RS04875	7.00	<0.0001	peptidase M20
OG1RF_RS13055	6.91	<0.0001	PTS sorbitol transporter subunit IIA

OG1RF_RS01710	6.83	<0.0001	5-keto-4-deoxyuronate isomerase
OG1RF_RS13070	6.78	<0.0001	transcriptional regulator
OG1RF_RS06150	6.74	<0.0001	molybdate ABC transporter permease subunit
OG1RF_RS00350	6.72	<0.0001	alkaline shock response membrane anchor protein AmaP
OG1RF_RS05245	6.69	<0.0001	endonuclease
OG1RF_RS06490	6.65	<0.0001	DUF871 domain-containing protein
OG1RF_RS07730	6.61	0.0031	DUF2188 domain-containing protein
OG1RF_RS12590	6.60	<0.0001	ABC transporter permease
OG1RF_RS01315	6.56	<0.0001	6-phospho-beta-glucosidase
OG1RF_RS13050	6.55	<0.0001	fructose-bisphosphate aldolase
OG1RF_RS05930	6.48	<0.0001	dihydrolipoyllysine-residue acetyltransferase
OG1RF_RS12300	6.36	0.0059	PTS mannose transporter subunit IID
OG1RF_RS05130	6.24	<0.0001	acetolactate decarboxylase
OG1RF_RS00495	6.24	0.0001	arginine deiminase
OG1RF_RS10930	6.22	0.0022	thiamine phosphate synthase
OG1RF_RS12280	6.22	<0.0001	4-hydroxy-2-oxoglutarate aldolase
OG1RF_RS06795	6.13	<0.0001	deoxyribodipyrimidine photo-lyase
OG1RF_RS13060	6.07	0.0003	PTS protein%2C glucitol/sorbitol%2C IIBC component
OG1RF_RS05125	6.05	<0.0001	acetolactate synthase AlsS
OG1RF_RS12695	6.03	<0.0001	DNA starvation/stationary phase protection protein
OG1RF_RS09960	6.01	<0.0001	sulfurtransferase-like selenium metabolism protein YedF
OG1RF_RS09995	5.93	<0.0001	purine permease
OG1RF_RS06000	5.93	<0.0001	hypothetical protein
OG1RF_RS12725	5.89	<0.0001	type II toxin-antitoxin system PemK/MazF family toxin
OG1RF_RS09130	5.89	<0.0001	gfo/Idh/MocA family oxidoreductase
OG1RF_RS05960	5.78	<0.0001	dihydroxyacetone kinase subunit L
OG1RF_RS05920	5.73	<0.0001	pyruvate dehydrogenase (acetyl-transferring) E1 component subunit alpha
OG1RF_RS06395	5.67	0.0119	PTS fructose transporter subunit IIA
OG1RF_RS06215	5.66	<0.0001	6-phospho-beta-glucosidase
OG1RF_RS13510	5.62	<0.0001	6S RNA
OG1RF_RS12285	5.59	0.0024	mannonate dehydratase
OG1RF_RS04740	5.49	0.0011	3-dehydro-L-gulonate-6-phosphate decarboxylase UlaD
OG1RF_RS09080	5.40	<0.0001	sugar ABC transporter substrate-binding protein
OG1RF_RS11790	5.39	0.0001	silent information regulator protein Sir2
OG1RF_RS01840	5.38	<0.0001	organic hydroperoxide resistance protein
OG1RF_RS06405	5.38	0.0001	ribulose-phosphate 3-epimerase
OG1RF_RS02175	5.36	<0.0001	type II CRISPR RNA-guided endonuclease Cas9
OG1RF_RS10460	5.34	0.0001	competence protein ComA
OG1RF_RS12730	5.32	<0.0001	hypothetical protein
OG1RF_RS05230	5.27	0.0003	hypothetical protein
OG1RF_RS06145	5.25	<0.0001	molybdate ABC transporter substrate-binding protein
OG1RF_RS03385	5.23	<0.0001	VOC family protein
OG1RF_RS10940	5.23	<0.0001	energy coupling factor transporter S component ThiW
OG1RF_RS09950	5.18	<0.0001	putative selenium-dependent hydroxylase accessory protein YqeC
OG1RF_RS06865	5.18	<0.0001	formate C-acetyltransferase
OG1RF_RS00540	5.18	0.0014	reactive intermediate/imine deaminase
OG1RF_RS04870	5.10	<0.0001	GntR family transcriptional regulator
OG1RF_RS07130	5.07	<0.0001	carboxylesterase
OG1RF_RS07165	5.04	<0.0001	lipoprotein

OG1RF_RS11570	5.03	0.0071	PTS fructose transporter subunit IID
OG1RF_RS02645	5.02	<0.0001	RNase adaptor protein RapZ
OG1RF_RS09110	4.99	0.0013	PTS cellobiose transporter subunit IIC
OG1RF_RS01550	4.97	<0.0001	serine protease
OG1RF_RS09135	4.92	0.0127	methylmalonate-semialdehyde dehydrogenase (CoA acylating)
OG1RF_RS05280	4.89	<0.0001	oxidoreductase
OG1RF_RS06740	4.87	<0.0001	NADH oxidase
OG1RF_RS13415	4.84	<0.0001	transfer-messenger RNA
OG1RF_RS02845	4.75	<0.0001	GlsB/YeaQ/YmgE family stress response membrane protein
OG1RF_RS09055	4.74	<0.0001	hypothetical protein
OG1RF_RS04745	4.73	0.0006	L-ribulose-5-phosphate 3-epimerase
OG1RF_RS10590	4.69	<0.0001	hypothetical protein
OG1RF_RS10895	4.69	<0.0001	ABC transporter ATP-binding protein
OG1RF_RS02225	4.65	<0.0001	N-acetyltransferase
OG1RF_RS10890	4.65	<0.0001	cobalt ABC transporter permease
OG1RF_RS02950	4.61	0.0006	hypothetical protein
OG1RF_RS06115	4.60	0.0002	molybdopterin molybdenumtransferase MoeA
OG1RF_RS09945	4.58	0.0005	hypothetical protein
OG1RF_RS12135	4.54	0.0004	ABC transporter permease
OG1RF_RS11715	4.53	<0.0001	PucR family transcriptional regulator
OG1RF_RS04560	4.49	<0.0001	isopeptide-forming domain-containing fimbrial protein
OG1RF_RS02540	4.46	<0.0001	hypothetical protein
OG1RF_RS11780	4.46	<0.0001	DinB family protein
OG1RF_RS03870	4.40	<0.0001	DUF3284 domain-containing protein
OG1RF_RS06730	4.34	0.0001	cysteine synthase A
OG1RF_RS02210	4.28	<0.0001	glycine/betaine ABC transporter permease
OG1RF_RS10025	4.27	0.0013	dihydropyrimidinase
OG1RF_RS09975	4.27	0.0002	molybdenum cofactor cytidylyltransferase
OG1RF_RS05955	4.26	<0.0001	dihydroxyacetone kinase subunit DhaK
OG1RF_RS02135	4.25	<0.0001	hypothetical protein
OG1RF_RS05865	4.21	<0.0001	sulfate ABC transporter ATP-binding protein
OG1RF_RS03130	4.20	0.0002	transcriptional regulator
OG1RF_RS01890	4.19	0.0001	superoxide dismutase [Fe]
OG1RF_RS08120	4.19	<0.0001	N-acetyltransferase
OG1RF_RS04645	4.18	<0.0001	lactate utilization protein C
OG1RF_RS04640	4.16	<0.0001	iron-sulfur cluster-binding protein
OG1RF_RS06450	4.16	<0.0001	type I glyceraldehyde-3-phosphate dehydrogenase
OG1RF_RS12305	4.10	0.0032	PTS sugar transporter subunit IIC
OG1RF_RS04400	4.09	<0.0001	universal stress protein
OG1RF_RS07125	4.09	<0.0001	ring-cleaving dioxygenase
OG1RF_RS05240	4.09	0.0010	acetylxylan esterase
OG1RF_RS11735	4.08	0.0129	hypothetical protein
OG1RF_RS06400	4.08	0.0008	PTS Fru family%2C IIC component
OG1RF_RS07135	4.04	0.0001	NADP-dependent oxidoreductase
OG1RF_RS11730	4.03	0.0039	DUF1116 domain-containing protein
OG1RF_RS12130	4.02	0.0005	ABC transporter permease
OG1RF_RS02295	4.02	0.0009	MurR/RpiR family transcriptional regulator
OG1RF_RS12145	3.92	<0.0001	ABC transporter ATP-binding protein
OG1RF_RS09965	3.89	0.0003	selenide%2C water dikinase SelD

OG1RF_RS10310	3.86	<0.0001	DUF697 domain-containing protein
OG1RF_RS02205	3.84	<0.0001	ABC transporter ATP-binding protein
OG1RF_RS06860	3.84	<0.0001	pyruvate formate lyase-activating protein
OG1RF_RS07115	3.83	0.0022	NAD(P)-dependent oxidoreductase
OG1RF_RS02445	3.81	<0.0001	hypothetical protein
OG1RF_RS01510	3.80	0.0036	MFS transporter
OG1RF_RS03160	3.79	0.0001	aldo/keto reductase
OG1RF_RS00320	3.76	0.0027	N-acetylmannosamine-6-phosphate 2-epimerase
OG1RF_RS12140	3.75	<0.0001	ABC transporter ATP-binding protein
OG1RF_RS06155	3.73	0.0009	molybdenum ABC transporter ATP-binding protein
OG1RF_RS13320	3.72	<0.0001	hypothetical protein
OG1RF_RS00615	3.72	0.0001	2-deoxyribose-5-phosphate aldolase
OG1RF_RS08955	3.71	0.0025	hypothetical protein
OG1RF_RS08300	3.71	<0.0001	sigma-54-dependent transcriptional regulator
OG1RF_RS12595	3.68	<0.0001	ABC transporter ATP-binding protein
OG1RF_RS09970	3.66	0.0002	aminotransferase class V-fold PLP-dependent enzyme
OG1RF_RS03955	3.65	0.0016	excinuclease ABC subunit UvrA
OG1RF_RS03150	3.65	<0.0001	copper-translocating P-type ATPase
OG1RF_RS06110	3.65	0.0001	formate dehydrogenase subunit alpha
OG1RF_RS13425	3.61	<0.0001	RNase P RNA component class B
OG1RF_RS04965	3.59	0.0006	YtxH domain-containing protein
OG1RF_RS00475	3.57	<0.0001	serine--tRNA ligase
OG1RF_RS04430	3.57	0.0005	VOC family protein
OG1RF_RS09115	3.55	0.0021	solute:sodium symporter family transporter
OG1RF_RS01705	3.51	0.0005	sugar kinase
OG1RF_RS10750	3.51	<0.0001	pyridine nucleotide-disulfide oxidoreductase
OG1RF_RS07490	3.51	<0.0001	YtxH domain-containing protein
OG1RF_RS12900	3.51	<0.0001	ATP-dependent Clp protease ATP-binding subunit
OG1RF_RS05310	3.49	0.0002	hypothetical protein
OG1RF_RS03510	3.47	0.0001	phosphate acetyltransferase
OG1RF_RS01855	3.46	0.0093	PTS mannose transporter subunit IID
OG1RF_RS04395	3.42	<0.0001	divalent metal cation transporter
OG1RF_RS13080	3.42	0.0019	NAD(P)-dependent oxidoreductase
OG1RF_RS02630	3.41	<0.0001	excinuclease ABC subunit UvrB
OG1RF_RS12500	3.40	0.0101	succinyl-diaminopimelate desuccinylase
OG1RF_RS01735	3.39	0.0032	TRAP transporter large permease
OG1RF_RS04555	3.39	<0.0001	isopeptide-forming domain-containing fimbrial protein
OG1RF_RS03900	3.37	0.0001	hypothetical protein
OG1RF_RS06880	3.37	0.0003	CoA-binding protein
OG1RF_RS00645	3.30	0.0022	ABC transporter permease
OG1RF_RS04750	3.22	0.0029	L-ribulose-5-phosphate 4-epimerase AraD
OG1RF_RS05670	3.21	<0.0001	flotillin family protein
OG1RF_RS02125	3.21	0.0001	YebC/PmpR family DNA-binding transcriptional regulator
OG1RF_RS10030	3.21	0.0002	putative selenate reductase subunit YgfK
OG1RF_RS06610	3.21	<0.0001	hypothetical protein
OG1RF_RS10755	3.20	0.0001	peroxiredoxin
OG1RF_RS11805	3.20	0.0001	serine protease
OG1RF_RS07590	3.16	0.0001	ribosomal subunit interface protein
OG1RF_RS08935	3.14	0.0001	PH domain-containing protein

OG1RF_RS08430	3.12	0.0001	universal stress protein
OG1RF_RS05950	3.11	0.0001	PTS-dependent dihydroxyacetone kinase phosphotransferase subunit DhaM
OG1RF_RS10470	3.06	0.0001	transcriptional regulator Spx
OG1RF_RS08420	3.06	0.0002	hypothetical protein
OG1RF_RS06520	3.03	0.0001	ECF transporter S component
OG1RF_RS02365	3.02	0.0001	ATP-dependent Clp protease ATP-binding subunit
OG1RF_RS10620	3.02	0.0008	beta-galactosidase subunit alpha
OG1RF_RS00345	3.02	0.0001	KR domain-containing protein
OG1RF_RS09575	3.01	0.0018	ChbG/HpnK family deacetylase
OG1RF_RS00670	3.00	0.0004	phosphopentomutase
OG1RF_RS04790	2.99	0.0001	lactoylglutathione lyase
OG1RF_RS00680	2.96	0.0002	purine-nucleoside phosphorylase
OG1RF_RS10450	2.93	0.0002	DsbA family protein
OG1RF_RS05275	2.92	0.0013	glycoside hydrolase family 1 protein
OG1RF_RS03805	2.90	0.0001	cell division protein DivIVA
OG1RF_RS05945	2.88	0.0004	glycerol dehydrogenase
OG1RF_RS06715	2.87	0.0001	transketolase
OG1RF_RS02220	2.87	0.0002	phosphoglucomutase
OG1RF_RS09175	2.86	0.0002	chaperone protein ClpB
OG1RF_RS10220	2.83	0.0003	N-acetyltransferase
OG1RF_RS04720	2.83	0.0106	L-ascorbate 6-phosphate lactonase
OG1RF_RS06185	2.83	0.0002	thioredoxin
OG1RF_RS02665	2.82	0.0002	thioredoxin
OG1RF_RS07395	2.82	0.0021	formate--tetrahydrofolate ligase
OG1RF_RS00610	2.81	0.0003	pyrimidine-nucleoside phosphorylase
OG1RF_RS05290	2.81	0.0003	iron-sulfur cluster biosynthesis family protein
OG1RF_RS08090	2.79	0.0006	ROK family protein
OG1RF_RS12600	2.78	0.0021	OsmC family peroxiredoxin
OG1RF_RS10660	2.77	0.0110	ABC-F type ribosomal protection protein Lsa(A)
OG1RF_RS11655	2.76	0.0004	PucR family transcriptional regulator
OG1RF_RS06330	2.76	0.0004	alpha/beta hydrolase
OG1RF_RS05385	2.76	0.0002	ATPase P
OG1RF_RS01465	2.74	0.0088	hypothetical protein
OG1RF_RS03450	2.74	0.0120	hypothetical protein
OG1RF_RS06140	2.72	0.0117	molybdenum cofactor biosynthesis protein MoaB
OG1RF_RS12575	2.71	0.0007	DNA-binding response regulator
OG1RF_RS01370	2.71	0.0004	aminopeptidase C
OG1RF_RS05260	2.70	0.0010	LacI family transcriptional regulator
OG1RF_RS00635	2.70	0.0006	ABC transporter ATP-binding protein
OG1RF_RS09930	2.69	0.0015	glutamate synthase (NADPH)%2C homotetrameric
OG1RF_RS00590	2.69	0.0037	esterase
OG1RF_RS01880	2.67	0.0026	PTS fructose transporter subunit IIA
OG1RF_RS10965	2.65	0.0006	DUF3042 domain-containing protein
OG1RF_RS00675	2.64	0.0007	purine-nucleoside phosphorylase
OG1RF_RS04635	2.63	0.0011	(Fe-S)-binding protein
OG1RF_RS02995	2.63	0.0035	sugar kinase
OG1RF_RS02320	2.63	0.0005	DUF402 domain-containing protein
OG1RF_RS09345	2.63	0.0006	Fe-S cluster assembly protein SufB
OG1RF_RS03255	2.63	0.0006	preprotein translocase subunit YajC

OG1RF_RS03120	2.63	0.0027	glyoxalase
OG1RF_RS07400	2.62	0.0046	cold shock domain-containing protein
OG1RF_RS11485	2.62	0.0006	ribose transporter RbsU
OG1RF_RS11700	2.62	0.0009	linear amide C-N hydrolase
OG1RF_RS04715	2.61	0.0015	DeoR/GlpR transcriptional regulator
OG1RF_RS01350	2.61	0.0069	copper-translocating P-type ATPase
OG1RF_RS02170	2.60	0.0023	hypothetical protein
OG1RF_RS02930	2.55	0.0015	gfo/Idh/MocA family oxidoreductase
OG1RF_RS09550	2.53	0.0044	permease
OG1RF_RS02525	2.49	0.0017	deoxynucleoside kinase
OG1RF_RS05850	2.48	0.0010	thioredoxin-disulfide reductase
OG1RF_RS11300	2.48	0.0013	ABC transporter substrate-binding protein
OG1RF_RS03780	2.47	0.0011	cell division protein FtsZ
OG1RF_RS00465	2.43	0.0037	L-serine ammonia-lyase%2C iron-sulfur-dependent%2C subunit beta
OG1RF_RS02650	2.42	0.0015	YvcK family protein
OG1RF_RS05705	2.41	0.0018	GntR family transcriptional regulator
OG1RF_RS06555	2.40	0.0019	30S ribosomal protein S1
OG1RF_RS06465	2.39	0.0036	PTS sugar transporter subunit IIC
OG1RF_RS06565	2.38	0.0022	HU family DNA-binding protein
OG1RF_RS11290	2.38	0.0026	2-hydroxyacid dehydrogenase
OG1RF_RS11330	2.37	0.0020	iron-sulfur cluster biosynthesis protein
OG1RF_RS03165	2.35	0.0024	DNA polymerase I
OG1RF_RS03225	2.35	0.0041	hypothetical protein
OG1RF_RS11595	2.34	0.0028	histidine phosphatase family protein
OG1RF_RS01940	2.34	0.0027	NrdH-redoxin
OG1RF_RS00620	2.34	0.0047	cytidine deaminase
OG1RF_RS02670	2.33	0.0026	ATP-dependent Clp protease proteolytic subunit
OG1RF_RS01970	2.33	0.0030	glyoxalase/bleomycin resistance/dioxygenase family protein
OG1RF_RS11910	2.32	0.0033	hypothetical protein
OG1RF_RS09580	2.32	0.0056	DUF47 domain-containing protein
OG1RF_RS02565	2.31	0.0029	rhodanese domain-containing protein
OG1RF_RS03190	2.31	0.0029	primosomal protein DnaI
OG1RF_RS00470	2.30	0.0043	L-serine ammonia-lyase%2C iron-sulfur-dependent%2C subunit alpha
OG1RF_RS09685	2.29	0.0033	inositol monophosphatase family protein
OG1RF_RS11210	2.29	0.0034	NAD(P)-dependent oxidoreductase
OG1RF_RS13340	2.29	0.0090	hypothetical protein
OG1RF_RS06125	2.28	0.0107	GTP 3'%2C8-cyclase MoaA
OG1RF_RS04665	2.27	0.0033	transcriptional regulator
OG1RF_RS05640	2.27	0.0037	N-acetylglucosamine-6-phosphate deacetylase
OG1RF_RS10225	2.27	0.0042	hypothetical protein
OG1RF_RS12260	2.27	0.0036	hypothetical protein
OG1RF_RS00705	2.27	0.0036	NADH-dependent alcohol dehydrogenase
OG1RF_RS00065	2.24	0.0046	50S ribosomal protein L9
OG1RF_RS08435	2.24	0.0038	acetate kinase
OG1RF_RS04040	2.23	0.0039	phosphogluconate dehydrogenase (NADP(+)-dependent%2C decarboxylating)
OG1RF_RS07530	2.22	0.0049	PspC domain-containing protein
OG1RF_RS11915	2.21	0.0076	hypothetical protein
OG1RF_RS09230	2.20	0.0060	gfo/Idh/MocA family oxidoreductase
OG1RF_RS12095	2.20	0.0054	glyoxalase

OG1RF_RS07260	2.19	0.0056	NAD(P)H-dependent oxidoreductase
OG1RF_RS06070	2.18	0.0052	peptidase T
OG1RF_RS12560	2.17	0.0098	cysteine hydrolase
OG1RF_RS10825	2.17	0.0130	anaerobic ribonucleoside-triphosphate reductase activating protein
OG1RF_RS07525	2.17	0.0059	phage holin family protein
OG1RF_RS03975	2.17	0.0089	nucleoside-diphosphate kinase
OG1RF_RS09360	2.16	0.0060	Fe-S cluster assembly protein SufD
OG1RF_RS04550	2.16	0.0051	VWA domain-containing protein
OG1RF_RS06210	2.16	0.0116	LacI family transcriptional regulator
OG1RF_RS06575	2.16	0.0059	tetratricopeptide repeat protein
OG1RF_RS07705	2.15	0.0067	membrane protein
OG1RF_RS01275	2.14	0.0054	beta-hydroxyacyl-ACP dehydratase
OG1RF_RS04710	2.13	0.0073	metallophosphoesterase
OG1RF_RS05270	2.13	0.0085	GntR family transcriptional regulator
OG1RF_RS12315	2.13	0.0109	2-hydroxyacid dehydrogenase
OG1RF_RS02655	2.13	0.0067	sporulation regulator WhiA
OG1RF_RS09500	2.12	0.0069	phospho-sugar mutase
OG1RF_RS02215	2.11	0.0084	arginine repressor
OG1RF_RS02065	2.11	0.0075	peptidase M48
OG1RF_RS02115	2.10	0.0072	M3B subfamily peptidase
OG1RF_RS06580	2.10	0.0087	hypothetical protein
OG1RF_RS05665	2.09	0.0096	hypothetical protein
OG1RF_RS12470	2.09	0.0110	damage-inducible protein A
OG1RF_RS08535	2.08	0.0080	cytochrome d ubiquinol oxidase subunit II
OG1RF_RS12380	2.08	0.0069	insulinase family protein
OG1RF_RS02660	2.08	0.0089	PEP phosphonomutase
OG1RF_RS02000	2.08	0.0093	hypothetical protein
OG1RF_RS10975	2.06	0.0135	DUF488 domain-containing protein
OG1RF_RS01470	2.04	0.0141	deaminase
OG1RF_RS07540	2.04	0.0098	phosphate transport system regulatory protein PhoU
OG1RF_RS00750	2.04	0.0138	class I SAM-dependent methyltransferase
OG1RF_RS11370	2.04	0.0107	UDP-N-acetylglucosamine 2-epimerase (non-hydrolyzing)
OG1RF_RS10195	2.04	0.0124	SsrA-binding protein
OG1RF_RS09780	2.04	0.0117	hypothetical protein
OG1RF_RS08500	2.03	0.0127	GntR family transcriptional regulator
OG1RF_RS03100	2.02	0.0114	ABC transporter permease
OG1RF_RS03835	2.01	0.0131	gfo/Idh/MocA family oxidoreductase
OG1RF_RS04975	0.51	0.0139	S-ribosylhomocysteine lyase
OG1RF_RS03140	0.50	0.0139	potassium transporter Kup
OG1RF_RS00585	0.50	0.0125	type I pantothenate kinase
OG1RF_RS12815	0.50	0.0132	geranylgeranyl pyrophosphate synthase
OG1RF_RS06850	0.49	0.0141	DUF1803 domain-containing protein
OG1RF_RS12230	0.49	0.0137	methionyl-tRNA formyltransferase
OG1RF_RS00925	0.49	0.0141	energy-coupling factor transporter transmembrane protein EcfT
OG1RF_RS07580	0.49	0.0135	peptide chain release factor 2
OG1RF_RS00120	0.49	0.0133	membrane protein
OG1RF_RS10175	0.49	0.0114	ATP synthase subunit B
OG1RF_RS02290	0.48	0.0130	tRNA (guanosine(46)-N7)-methyltransferase TrmB
OG1RF_RS07100	0.48	0.0121	YdcF family protein

OG1RF_RS08820	0.48	0.0101	hypothetical protein
OG1RF_RS06630	0.48	0.0143	chorismate synthase
OG1RF_RS09810	0.47	0.0130	methionine ABC transporter ATP-binding protein
OG1RF_RS10410	0.47	0.0083	tRNA (uridine(34)/cytosine(34)/5-carboxymethylaminomethyluridine(34)-2'-O)-methyltransferase TrmL
OG1RF_RS01645	0.47	0.0112	FAD-binding oxidoreductase
OG1RF_RS10785	0.47	0.0092	D-alanyl-lipoteichoic acid biosynthesis protein DltD
OG1RF_RS10415	0.47	0.0139	methyltransferase domain-containing protein
OG1RF_RS10060	0.47	0.0066	inosine-uridine preferring nucleoside hydrolase
OG1RF_RS10155	0.47	0.0076	ATP synthase subunit beta
OG1RF_RS00720	0.47	0.0090	ribose-5-phosphate isomerase
OG1RF_RS00075	0.47	0.0080	adenylosuccinate synthetase
OG1RF_RS10120	0.46	0.0065	FMN-dependent NADH-azoreductase
OG1RF_RS08970	0.46	0.0077	nucleoid-associated protein
OG1RF_RS01055	0.46	0.0086	tRNA dihydrouridine synthase DusB
OG1RF_RS00895	0.46	0.0133	50S ribosomal protein L17
OG1RF_RS08760	0.46	0.0073	epimerase
OG1RF_RS01560	0.46	0.0059	AI-2E family transporter
OG1RF_RS01035	0.46	0.0105	tRNA lysidine(34) synthetase TilS
OG1RF_RS03035	0.46	0.0085	alanine racemase
OG1RF_RS13015	0.46	0.0100	DUF951 domain-containing protein
OG1RF_RS10910	0.46	0.0087	MFS transporter
OG1RF_RS01780	0.46	0.0058	peptide MFS transporter
OG1RF_RS11345	0.46	0.0076	sensor histidine kinase
OG1RF_RS03320	0.45	0.0053	ABC transporter ATP-binding protein
OG1RF_RS11255	0.45	0.0065	hypothetical protein
OG1RF_RS11350	0.45	0.0074	membrane protein
OG1RF_RS10870	0.45	0.0058	thymidylate kinase
OG1RF_RS01975	0.45	0.0052	cardiolipin synthase
OG1RF_RS10445	0.45	0.0072	CYTH domain-containing protein
OG1RF_RS09830	0.45	0.0063	FtsW/RodA/SpoVE family cell cycle protein
OG1RF_RS10835	0.45	0.0076	iron export ABC transporter permease subunit FetB
OG1RF_RS06035	0.45	0.0065	hypothetical protein
OG1RF_RS00180	0.45	0.0129	YafY family transcriptional regulator
OG1RF_RS11220	0.44	0.0080	glycosyl transferase family 1
OG1RF_RS06645	0.44	0.0078	shikimate kinase
OG1RF_RS01885	0.44	0.0052	DUF1189 domain-containing protein
OG1RF_RS05010	0.44	0.0045	TIGR01906 family membrane protein
OG1RF_RS09190	0.44	0.0055	5-(carboxyamino)imidazole ribonucleotide synthase
OG1RF_RS08665	0.44	0.0049	hypothetical protein
OG1RF_RS01600	0.44	0.0059	nitroreductase family protein
OG1RF_RS12225	0.44	0.0038	16S rRNA (cytosine(967)-C(5))-methyltransferase RsmB
OG1RF_RS11450	0.43	0.0038	tRNA 4-thiouridine(8) synthase ThiI
OG1RF_RS11430	0.43	0.0076	TVP38/TMEM64 family protein
OG1RF_RS05630	0.43	0.0054	hypothetical protein
OG1RF_RS12800	0.43	0.0024	NAD(P)/FAD-dependent oxidoreductase
OG1RF_RS02700	0.43	0.0038	glycerophosphoryl diester phosphodiesterase
OG1RF_RS03735	0.43	0.0037	division/cell wall cluster transcriptional repressor MraZ
OG1RF_RS11215	0.43	0.0035	glycosyltransferase family 4 protein
OG1RF_RS04815	0.42	0.0028	DUF1934 domain-containing protein

OG1RF_RS10650	0.42	0.0081	50S ribosomal protein L1
OG1RF_RS04820	0.42	0.0018	DNA-directed RNA polymerase subunit delta
OG1RF_RS05525	0.42	0.0025	riboflavin biosynthesis protein RibF
OG1RF_RS06635	0.42	0.0143	prephenate dehydrogenase
OG1RF_RS06415	0.41	0.0114	lysozyme family protein
OG1RF_RS00910	0.41	0.0098	amidohydrolase
OG1RF_RS03645	0.41	0.0111	ribosomal-processing cysteine protease Prp
OG1RF_RS11280	0.41	0.0025	ferredoxin--NADP(+) reductase
OG1RF_RS07630	0.41	0.0038	3-oxoacyl-ACP reductase
OG1RF_RS03315	0.41	0.0017	ABC transporter permease
OG1RF_RS07450	0.41	0.0068	DUF1801 domain-containing protein
OG1RF_RS00745	0.41	0.0092	bifunctional hydroxymethylpyrimidine kinase/phosphomethylpyrimidine kinase
OG1RF_RS09505	0.40	0.0037	PLP-dependent aminotransferase family protein
OG1RF_RS10400	0.40	0.0015	ATP-dependent RecD-like DNA helicase
OG1RF_RS09265	0.40	0.0014	PolC-type DNA polymerase III
OG1RF_RS03275	0.40	0.0044	phosphomevalonate kinase
OG1RF_RS09900	0.40	0.0023	peptide chain release factor N(5)-glutamine methyltransferase
OG1RF_RS12660	0.40	0.0115	PTS mannose transporter subunit IID
OG1RF_RS10075	0.40	0.0024	serine hydrolase
OG1RF_RS13175	0.40	0.0029	DNA-binding response regulator
OG1RF_RS09795	0.40	0.0021	isoprenyl transferase
OG1RF_RS02785	0.40	0.0017	TIGR01212 family radical SAM protein
OG1RF_RS03370	0.39	0.0085	SulP family inorganic anion transporter
OG1RF_RS11025	0.39	0.0076	FUSC family protein
OG1RF_RS12340	0.39	0.0020	CDP-diacylglycerol--glycerol-3-phosphate 3-phosphatidyltransferase
OG1RF_RS06350	0.39	0.0009	metal-sulfur cluster assembly factor
OG1RF_RS11385	0.39	0.0054	ABC transporter ATP-binding protein
OG1RF_RS08885	0.39	0.0020	glycosyltransferase family 2 protein
OG1RF_RS05200	0.39	0.0016	hypothetical protein
OG1RF_RS02825	0.39	0.0118	amino acid ABC superfamily ATP-binding cassette transporter -binding protein
OG1RF_RS06790	0.39	0.0037	catalase
OG1RF_RS04945	0.39	0.0007	50S ribosomal protein L31 type B
OG1RF_RS12090	0.39	0.0013	YitT family protein
OG1RF_RS01040	0.38	0.0012	hypoxanthine-guanine phosphoribosyltransferase
OG1RF_RS10760	0.38	0.0007	O-methyltransferase
OG1RF_RS13000	0.38	0.0012	membrane protein
OG1RF_RS09640	0.38	0.0032	16S rRNA (guanine(966)-N(2))-methyltransferase RsmD
OG1RF_RS00940	0.38	0.0006	branched-chain amino acid transport system II carrier protein
OG1RF_RS04050	0.38	0.0007	sensor histidine kinase
OG1RF_RS08105	0.38	0.0010	GTP-binding protein
OG1RF_RS01565	0.37	0.0014	membrane protein
OG1RF_RS12665	0.37	0.0021	PRD domain-containing protein
OG1RF_RS02780	0.37	0.0009	rRNA methyltransferase
OG1RF_RS02120	0.37	0.0099	membrane protein
OG1RF_RS05070	0.36	0.0123	threonine/serine exporter
OG1RF_RS09675	0.36	0.0012	DNA helicase RecQ
OG1RF_RS06725	0.36	0.0006	N-acetylmuramoyl-L-alanine amidase
OG1RF_RS07050	0.36	0.0038	ribosome biogenesis GTPase YlqF
OG1RF_RS09570	0.36	0.0004	undecaprenyl-diphosphatase

OG1RF_RS09695	0.36	0.0063	voltage-gated chloride channel protein
OG1RF_RS10595	0.36	0.0055	LytR family transcriptional regulator
OG1RF_RS09740	0.36	0.0027	ribosomal-protein-alanine N-acetyltransferase
OG1RF_RS03230	0.36	0.0102	pyridoxal phosphate-dependent aminotransferase
OG1RF_RS03280	0.36	0.0012	diphosphomevalonate decarboxylase
OG1RF_RS10270	0.36	0.0004	co-chaperone GroES
OG1RF_RS04600	0.36	0.0013	ABC transporter ATP-binding protein
OG1RF_RS09405	0.36	0.0002	cytochrome oxidase biogenesis protein OxaA
OG1RF_RS12810	0.35	0.0025	heptaprenyl diphosphate synthase subunit I
OG1RF_RS11055	0.35	0.0003	tautomerase
OG1RF_RS10800	0.35	0.0004	D-alanine--poly(phosphoribitol) ligase subunit 1
OG1RF_RS02110	0.35	0.0050	MATE family efflux transporter
OG1RF_RS11920	0.35	0.0011	class A sortase
OG1RF_RS04535	0.35	0.0008	thioredoxin
OG1RF_RS06550	0.34	0.0003	cytidylate kinase
OG1RF_RS02620	0.34	0.0002	amino acid ABC transporter ATP-binding protein
OG1RF_RS08890	0.34	0.0010	glycosyl transferase family 2
OG1RF_RS11425	0.34	0.0003	hypothetical protein
OG1RF_RS08065	0.34	0.0002	enoyl-CoA hydratase
OG1RF_RS01445	0.34	0.0074	sensor histidine kinase
OG1RF_RS08810	0.34	0.0009	glycosyl transferase family 2
OG1RF_RS07280	0.34	0.0048	DUF523 domain-containing protein
OG1RF_RS06535	0.34	0.0014	hypothetical protein
OG1RF_RS06505	0.33	0.0003	segregation/condensation protein A
OG1RF_RS08520	0.33	0.0004	polyprenyl synthetase family protein
OG1RF_RS12390	0.33	0.0001	large conductance mechanosensitive channel protein MscL
OG1RF_RS12605	0.33	0.0025	30S ribosomal protein S14
OG1RF_RS09465	0.33	0.0006	transcriptional repressor
OG1RF_RS02510	0.33	0.0002	amidase
OG1RF_RS03270	0.33	0.0041	type 2 isopentenyl-diphosphate Delta-isomerase
OG1RF_RS09680	0.33	0.0058	translational GTPase TypA
OG1RF_RS00410	0.33	0.0090	hypothetical protein
OG1RF_RS02745	0.32	0.0024	TetR family transcriptional regulator
OG1RF_RS10330	0.32	0.0001	GntP family permease
OG1RF_RS09565	0.32	0.0002	PTS system protein%2C IIA component
OG1RF_RS11520	0.31	<0.0001	PRD domain-containing protein
OG1RF_RS00455	0.31	0.0001	hypothetical protein
OG1RF_RS10655	0.31	0.0026	50S ribosomal protein L11
OG1RF_RS04785	0.31	0.0002	gamma-glutamyl-gamma-aminobutyrate hydrolase
OG1RF_RS12690	0.31	<0.0001	50S ribosomal protein L13
OG1RF_RS00255	0.31	0.0001	4-diphosphocytidyl-2C-methyl-D-erythritol kinase
OG1RF_RS02330	0.31	0.0001	AI-2E family transporter
OG1RF_RS13085	0.30	<0.0001	tRNA uridine-5-carboxymethylaminomethyl(34) synthesis enzyme MnmG
OG1RF_RS08240	0.30	0.0001	Bcr/CflA family drug resistance efflux transporter
OG1RF_RS08845	0.30	0.0003	ABC transporter permease
OG1RF_RS12490	0.30	0.0121	YccF domain-containing protein
OG1RF_RS03310	0.30	<0.0001	ABC transporter permease
OG1RF_RS09745	0.30	0.0002	ribosomal-protein-alanine N-acetyltransferase
OG1RF_RS05215	0.30	0.0060	metallophosphoesterase

OG1RF_RS08330	0.30	<0.0001	triose-phosphate isomerase
OG1RF_RS06845	0.30	0.0006	DUF2179 domain-containing protein
OG1RF_RS06980	0.29	0.0001	ABC transporter ATP-binding protein
OG1RF_RS13090	0.29	<0.0001	tRNA uridine-5-carboxymethylaminomethyl(34) synthesis GTPase MnmE
OG1RF_RS02790	0.29	0.0001	phosphatase PAP2 family protein
OG1RF_RS11990	0.29	<0.0001	30S ribosomal protein S4
OG1RF_RS12025	0.29	<0.0001	DUF1054 domain-containing protein
OG1RF_RS01595	0.29	0.0009	MarR family transcriptional regulator
OG1RF_RS08335	0.29	<0.0001	phosphoglycerate kinase
OG1RF_RS06510	0.29	0.0001	SMC-Scp complex subunit ScpB
OG1RF_RS10245	0.29	0.0029	GtrA family protein
OG1RF_RS06625	0.28	<0.0001	3-dehydroquinate synthase
OG1RF_RS12785	0.28	0.0009	1%2C4-dihydroxy-2-naphthoate polyprenyltransferase
OG1RF_RS00305	0.28	0.0047	Holliday junction branch migration protein RuvA
OG1RF_RS01425	0.28	0.0003	HAD family phosphatase
OG1RF_RS04625	0.28	0.0029	hypothetical protein
OG1RF_RS10390	0.28	0.0003	QueT transporter family protein
OG1RF_RS03515	0.28	0.0003	tRNA (adenosine(37)-N6)-threonylcarbamoyltransferase complex ATPase subunit type 1 TsaE
OG1RF_RS00525	0.28	0.0139	ArsR family transcriptional regulator
OG1RF_RS07285	0.28	0.0001	DNA-binding response regulator
OG1RF_RS03285	0.28	0.0036	mevalonate kinase
OG1RF_RS03330	0.28	0.0001	transposase
OG1RF_RS13190	0.28	0.0001	ribonuclease P protein component
OG1RF_RS03410	0.28	<0.0001	amino acid permease
OG1RF_RS12250	0.27	<0.0001	guanylate kinase
OG1RF_RS02905	0.27	<0.0001	16S rRNA pseudouridine(516) synthase
OG1RF_RS09860	0.27	0.0110	branched-chain amino acid ABC transporter permease
OG1RF_RS06370	0.27	0.0010	transcription antiterminator BglG
OG1RF_RS02675	0.27	<0.0001	hypothetical protein
OG1RF_RS08735	0.27	0.0134	MerR family transcriptional regulator
OG1RF_RS11785	0.27	<0.0001	dicarboxylate/amino acid:cation symporter
OG1RF_RS08025	0.27	<0.0001	50S ribosomal protein L19
OG1RF_RS01580	0.27	<0.0001	DUF979 domain-containing protein
OG1RF_RS04935	0.26	<0.0001	UDP-N-acetylglucosamine 1-carboxyvinyltransferase
OG1RF_RS10405	0.26	0.0001	histidine phosphatase family protein
OG1RF_RS08560	0.25	0.0014	TetR/AcrR family transcriptional regulator
OG1RF_RS11895	0.25	0.0002	peptidase C39 family protein
OG1RF_RS04620	0.25	0.0035	hypothetical protein
OG1RF_RS09240	0.25	<0.0001	pyridoxal phosphate-dependent aminotransferase
OG1RF_RS06985	0.25	0.0002	iron ABC transporter permease
OG1RF_RS08895	0.25	0.0004	glycosyltransferase family 2 protein
OG1RF_RS00950	0.25	<0.0001	TIGR00730 family Rossman fold protein
OG1RF_RS09530	0.25	<0.0001	guanine deaminase
OG1RF_RS05520	0.25	<0.0001	tRNA pseudouridine(55) synthase TruB
OG1RF_RS06540	0.25	<0.0001	ATP-dependent DNA helicase RecQ
OG1RF_RS07715	0.25	0.0010	peptide ABC transporter substrate-binding protein
OG1RF_RS05055	0.25	0.0001	permease
OG1RF_RS10795	0.25	0.0002	D-alanyl-lipoteichoic acid biosynthesis protein DltB
OG1RF_RS09790	0.24	<0.0001	phosphatidate cytidylyltransferase

OG1RF_RS09520	0.24	<0.0001	GMP reductase
OG1RF_RS02920	0.24	<0.0001	uridine kinase
OG1RF_RS03660	0.24	0.0003	metallophosphoesterase
OG1RF_RS06620	0.24	0.0004	3-deoxy-7-phosphoheptulonate synthase
OG1RF_RS09250	0.24	<0.0001	DNA polymerase III subunit epsilon
OG1RF_RS07105	0.24	0.0002	conjugal transfer protein TraX
OG1RF_RS10690	0.24	0.0124	MurR/RpiR family transcriptional regulator
OG1RF_RS08000	0.23	<0.0001	DeoR/GlpR transcriptional regulator
OG1RF_RS03235	0.23	<0.0001	amino acid ABC transporter ATP-binding protein
OG1RF_RS08340	0.23	<0.0001	type I glyceraldehyde-3-phosphate dehydrogenase
OG1RF_RS12790	0.23	0.0002	FAD:protein FMN transferase
OG1RF_RS10540	0.23	0.0005	tRNA1(Val) (adenine(37)-N6)-methyltransferase
OG1RF_RS10905	0.23	0.0001	membrane protein
OG1RF_RS13030	0.23	<0.0001	ribosomal RNA small subunit methyltransferase G
OG1RF_RS00015	0.23	<0.0001	chromosomal replication initiator protein DnaA
OG1RF_RS08915	0.22	<0.0001	flavodoxin
OG1RF_RS10840	0.22	<0.0001	ABC transporter ATP-binding protein
OG1RF_RS09835	0.22	0.0012	hypothetical protein
OG1RF_RS11465	0.21	0.0001	NCS2 family permease
OG1RF_RS12085	0.21	0.0004	cysteine hydrolase
OG1RF_RS01210	0.21	<0.0001	PTS beta-glucoside transporter subunit EIIBCA
OG1RF_RS08990	0.21	0.0001	FUSC family protein
OG1RF_RS12995	0.20	<0.0001	IMP dehydrogenase
OG1RF_RS09385	0.20	<0.0001	30S ribosomal protein S2
OG1RF_RS03490	0.20	0.0001	N-acetyltransferase
OG1RF_RS13680	0.20	<0.0001	hypothetical protein
OG1RF_RS11010	0.20	<0.0001	DUF805 domain-containing protein
OG1RF_RS02625	0.20	<0.0001	glutamine ABC transporter substrate-binding protein GlnH
OG1RF_RS09040	0.20	0.0084	ABC transporter ATP-binding protein
OG1RF_RS03240	0.20	<0.0001	glutamate ABC transporter permease
OG1RF_RS10115	0.19	<0.0001	transcription antiterminator BglG
OG1RF_RS00190	0.19	0.0001	glutamate 5-kinase
OG1RF_RS08855	0.18	<0.0001	DUF2304 domain-containing protein
OG1RF_RS12030	0.18	<0.0001	N-acetyltransferase
OG1RF_RS08480	0.18	<0.0001	amino acid permease
OG1RF_RS08365	0.18	<0.0001	hydroxymethylpyrimidine/phosphomethylpyrimidine kinase
OG1RF_RS02710	0.18	0.0059	cold-shock protein
OG1RF_RS11185	0.18	<0.0001	enoyl-[acyl-carrier-protein] reductase FabK
OG1RF_RS00115	0.17	<0.0001	DUF956 domain-containing protein
OG1RF_RS08695	0.17	<0.0001	heme ABC transporter ATP-binding protein
OG1RF_RS04595	0.17	0.0005	adhesin
OG1RF_RS00905	0.17	<0.0001	DUF819 domain-containing protein
OG1RF_RS10485	0.17	<0.0001	haloacid dehalogenase
OG1RF_RS02640	0.17	<0.0001	hypothetical protein
OG1RF_RS01980	0.16	0.0065	Tyrosine--tRNA ligase 1
OG1RF_RS06995	0.16	<0.0001	glycerol-3-phosphate acyltransferase
OG1RF_RS01575	0.16	<0.0001	DUF969 domain-containing protein
OG1RF_RS01790	0.16	<0.0001	peptidase M23
OG1RF_RS11980	0.16	<0.0001	DNA alkylation repair protein

OG1RF_RS09245	0.16	<0.0001	peptidase
OG1RF_RS08850	0.15	<0.0001	hypothetical protein
OG1RF_RS00445	0.15	0.0001	formate/nitrite transporter
OG1RF_RS01050	0.15	<0.0001	redox-regulated molecular chaperone Hsp33
OG1RF_RS08690	0.14	<0.0001	energy-coupling factor transporter transmembrane protein EcfT
OG1RF_RS00965	0.14	<0.0001	hypothetical protein
OG1RF_RS12510	0.14	0.0008	sigma-70 family RNA polymerase sigma factor
OG1RF_RS12060	0.14	0.0001	iron ABC transporter permease
OG1RF_RS02815	0.14	0.0009	DUF3955 domain-containing protein
OG1RF_RS02430	0.13	<0.0001	ClC family H(+)/Cl(-) exchange transporter
OG1RF_RS11695	0.13	<0.0001	SulP family inorganic anion transporter
OG1RF_RS03085	0.13	<0.0001	cation transporter
OG1RF_RS09525	0.13	<0.0001	purine permease
OG1RF_RS06615	0.12	<0.0001	shikimate dehydrogenase
OG1RF_RS07720	0.12	0.0003	cupin domain-containing protein
OG1RF_RS06075	0.12	0.0006	DUF1294 domain-containing protein
OG1RF_RS04590	0.11	0.0004	DUF898 domain-containing protein
OG1RF_RS08700	0.11	<0.0001	DUF3816 family protein
OG1RF_RS11140	0.11	0.0001	YqeG family HAD IIIA-type phosphatase
OG1RF_RS11985	0.10	<0.0001	formate/nitrite transporter
OG1RF_RS02695	0.10	0.0131	hypothetical protein
OG1RF_RS04000	0.10	<0.0001	NCS2 family permease
OG1RF_RS02425	0.09	<0.0001	DeoR/GlpR transcriptional regulator
OG1RF_RS01985	0.09	0.0025	tyrosine decarboxylase
OG1RF_RS12655	0.09	<0.0001	PTS mannose/fructose/sorbose/N-acetylgalactosamine transporter subunit IIC
OG1RF_RS00095	0.08	0.0001	PTS fructose transporter subunit IIB
OG1RF_RS01685	0.08	0.0002	MFS transporter
OG1RF_RS01205	0.07	<0.0001	PRD domain-containing protein
OG1RF_RS01590	0.07	<0.0001	Na+/H+ antiporter NhaC
OG1RF_RS01680	0.07	<0.0001	DUF4811 domain-containing protein
OG1RF_RS09200	0.07	<0.0001	purine permease
OG1RF_RS03405	0.06	<0.0001	glucose transporter GlcU
OG1RF_RS05175	0.06	<0.0001	amidohydrolase
OG1RF_RS02345	0.06	0.0117	DUF3290 domain-containing protein
OG1RF_RS01990	0.06	0.0005	amino acid permease
OG1RF_RS12650	0.05	<0.0001	PTS mannose/fructose/sorbose transporter subunit IIB
OG1RF_RS00110	0.05	<0.0001	PTS mannose transporter subunit IID
OG1RF_RS10305	0.05	0.0014	glycine/betaine ABC transporter permease
OG1RF_RS01995	0.05	<0.0001	Na+/H+ antiporter NhaC
OG1RF_RS00100	0.05	<0.0001	PTS mannose transporter subunit IIAB
OG1RF_RS09205	0.05	<0.0001	xanthine phosphoribosyltransferase
OG1RF_RS10300	0.04	0.0006	glycine betaine/L-proline ABC transporter ATP-binding protein
OG1RF_RS00400	0.04	0.0138	MFS transporter
OG1RF_RS05170	0.04	<0.0001	adenine deaminase
OG1RF_RS12645	0.03	<0.0001	PTS mannose transporter subunit IIA
OG1RF_RS00105	0.03	<0.0001	PTS mannose/fructose/sorbose transporter subunit IIC
OG1RF_RS02820	0.03	<0.0001	hypothetical protein
OG1RF_RS05165	0.03	0.0002	ABC transporter substrate-binding protein
OG1RF_RS02415	0.03	<0.0001	PTS fructose transporter subunit IIC

OG1RF_RS10110	0.03	<0.0001	PTS beta-glucoside transporter subunit EIIBCA
OG1RF_RS02420	0.02	<0.0001	1-phosphofructokinase
OG1RF_RS05025	0.02	<0.0001	MIP family channel protein
OG1RF_RS05155	0.02	0.0001	ABC transporter permease
OG1RF_RS05160	0.02	0.0001	ABC transporter ATP-binding protein
OG1RF_RS13565	0.02	0.0003	hypothetical protein

[a]The DEGs of RNA-sequencing were defined by change ratio ≥ 2 and $p < 0.05$.
[b]The *p*-values for the DEGs of RNA-sequencing.

Table S3. The reads and reference genome comparison statistics of RNA-sequencing.

Sample name	EF16_1	EF16_2	EF16_3	EF16-O2_1	EF16-O2_2	EF16-O2_3
**Total reads**	13298542	11193758	9321776	13840844	14227328	14246934
**Total mapped[a]**	12769019 (96.02%)	10842029 (96.86%)	8998740 (96.53%)	13101150 (94.66%)	13447696 (94.52%)	13661875 (95.89%)
**Multiple mapped**	230574 (1.73%)	157872 (1.41%)	134491 (1.44%)	167526 (1.21%)	254088 (1.79%)	241196 (1.69%)
**Uniquely mapped**	12538445 (94.28%)	10684157 (95.45%)	8864249 (95.09%)	12933624 (93.45%)	13193608 (92.73%)	13420679 (94.2%)
**Unmapped**	529523 (3.98%)	351729 (3.14%)	323036 (3.47%)	739694 (5.34%)	779632 (5.48%)	585059 (4.11%)

[a]Total mapped: Number of reads that can be mapped to the reference genome.
Multiple mapped: Number of reads with multiple alignment positions on the reference genome.
Uniquely mapped: Number of reads with unique alignment positions on the reference genome.
Unmapped: Number of reads that cannot be mapped to the reference genome.

Table S4. Transcriptional expression levels of six candidate genes in ten *E. faecalis* clinical isolates for transformation.

**Isolate**	**qRT-PCR expression ratio (strain/ OG1RF)**					
	**RS00630**	**RS02205**	**RS06145**	**RS06880**	**RS11485**	**RS12140**
EF16C105	3.69	0.86	0.56	0.83	2.12	0.20
EF16C2	1.24	0.01	0.97	5.37	12.93	1.64
EF16C283	2.29	0.88	6.52	1.92	6.02	0.29
EF16C284	0.20	0.52	1.05	0.19	0.64	7.89
EF16C286	0.29	3.74	0.76	0.28	1.50	2.02
EF16C387	1.56	0.27	3.03	1.81	6.53	0.64
EF16C39	1.94	0.54	1.16	1.06	3.44	1.48
EF16C4	1.40	0.36	0.38	2.06	10.48	1.33
EF16C40	1.75	1.29	2.76	2.27	11.10	2.24
EF16C5	0.93	0.48	0.67	1.41	4.35	8.58

Relative expressions of six candidate genes in OMC-sensitive isolates were determined by qRT-PCR analysis. The housekeeping gene *recA* was used as the endogenous reference gene. The *E. faecalis* OG1RF strain was used as the reference strain (expression = 1.0).

Table S5. Transformation of six candidate genes in clinical *E. faecalis* isolates.

Candidate gene	Strain1	Strain 2	Strain 3
RS00630	EF16C2	EF16C105	EF16C284
RS02205	EF16C2	EF16C105	EF16C39
RS06145	EF16C2	EF16C105	EF16C283
RS06880	EF16C2	EF16C105	EF16C284
RS11485	EF16C2	EF16C105	EF16C39
RS12140	EF16C105	EF16C39	EF16C40

Table S6. Homology analysis of *E. faecalis* OG1RF_RS00630 encoding protein (AEA92812.1).

Strain	*E. faecalis* OG1RF AEA92812.1			
	**GenBank ID**	**No. amino acids**	**Identical %**	**Description or predicted function**
E. faecalis ATCC 29212	AIL03342.1	361	96.3	Basic membrane family protein
E. faecalis 62	ADX78833.1	361	96.3	Ribonucleoside ABC transporter family protein
E. faecalis V583	AAO80051.1	361	96.3	Basic membrane protein family
E. faecalis DENG1	AHI39275.1	361	96.3	Basic membrane protein family
E. faecium 13	EPH67717.1	392	75.5	Basic membrane protein
E. faecium 505	EJY46432.1	373	71.6	Basic membrane protein
E. casseliflavus	WP_016609144.1	372	75.4	BMP family ABC transporter substrate-binding protein
E. canintestini	WP_071864055.1	373	72.6	BMP family ABC transporter substrate-binding protein
S. aureus	SUL86877.1	347	62.5	Purine nucleoside receptor A
S. pneumoniae	CCM08442.1	350	60.9	putative lipoprotein
S. agalactiae	WP_065736824.1	349	62	BMP family ABC transporter substrate-binding protein
K. pneumoniae	WP_116290355.1	88	16.3	BMP family ABC transporter substrate-binding protein
E. coli	WP_116834533.1	112	17.9	BMP family ABC transporter substrate-binding protein
A. baumannii	RJN76617.1	55	5.1	3-hydroxyacyl-CoA dehydrogenase

Table S7. Primers used for TET-specific resistance genes amplification.

**Target gene**	**Primer**	**Primer sequence (5'-3')**	**Amplicon size (bp)**	**Reference**
tet(M)	tet(M)-F	CAATACAATAGGAGCAAGC	974	This study
	tet(M)-R	CGAACAAGAGGAAAGCATAAG		
tet(L)	tet(L)-F	GTAACCAGCCAACTAATGAC	908	This study
	tet(L)-R	TTGGATCGATAGTAGCC		
tet(K)	tet(K)-F	TCGATAGGAACAGCAGTA	169	(1)
	tet(K)-R	CAGCAGATCCTACTCCTT		
tet(S)	tet(S)-F	TACCTCCATTTGGACCTCAC	1050	This study
	tet(S)-R	GAACGCCAGAGAGGTATT		
tet(W)	tet(W)-F	GAGAGCCTGCTATATGCCAGC	168	(1)
	tet(W)-R	GGGCGTATCCACAATGTTAAC		
tet(O)	tet(O)-F	AACTTAGGCATTCTGGCTCAC	515	(1)
	tet(O)-R	TCCCACTGTTCCATATCGTCA		
tet(U)	tet(U)-F	CAAAAGAAATCGATACGTGG	294	(2)
	tet(U)-R	CGTCTGCAGATTCCTTAAAAGTC		

Table S8. Primers used for MLST determination in *E. faecalis*.

Target	Primer	Primer sequence (5'-3')	Amplicon size (bp)	Reference
gdh[a]	gdh-F	GGCGCACTAAAAGATATGGT	530	(3)
	gdh-R	CCAAGATTGGGCAACTTCGTCCCA		
gyd	gyd-F	CAAACTGCTTAG CTCCAATGGC	395	(3)
	gyd-R	CATTTCGTTGTCATACCAAGC		
pstS	pstS-F	CGGAACAGGACTTTCGC	583	(3)
	pstS-R	ATTTACATCACGTTCTACTTGC		
gki	gki-F	GATTTTGTGGGAATTGGTATGG	438	(3)
	gki-R	ACCATTAAAGCAAAATGATCGC		
aroE	aroE-F	TGGAAAACTTTACGGAGACAGC	459	(3)
	aroE-R	GTCCTG TCCATTGTTCAAAAGC		
xpt	xpt-F	AAAATGATGGCCGTGTATTAGG,	456	(3)
	xpt-R	AACGTCACCGTTCCTTCACTTA		
yqiL	yqiL-F	CAGCTTAAGTCAAG TAAGTGCCG	436	(3)
	yqiL-R	GAATATCCCTTCTGCTTGTGCT		

[a]*Gdh*: glucose-6-phosphate dehydrogenase; *gyd*: glyceraldehyde-3-phosphate dehydrogenase; *pstS*: phosphate-ATP binding cassette transporter; *gki*: glucokinase; *aroE*: shikimate 5-dehydrogenase; *xpt*: xanthine phosphoribosyltransferase; *yqiI*: acetyl-CoA acetyltransferase.

Table S9. Primers used for amplification of 30S ribosomal subunits of *E. faecalis.*

Target gene	Primer	Primer sequence (5'-3')	Amplicon size (bp)	Reference
RR1[a]	RR1-F	CCGGAGGATTAGGTATTGGGATTGA	2356	This study
	RR1-R	CTCAAAACTGAACAAAGTAAAGACG		
RR2	RR2-F	CTGAACAAGTGGACACCCAA	2767	This study
	RR2-R	CTCAAAACTGAACAAAGTAAAGACG		
RR3	RR3-F	CGAATAGAAATGCTTGGGAAC	2995	This study
	RR3-R	CTCAAAACTGAACAAAGTAAAGACG		
RR4	RR4-F	GTAGCCCAAGAAGAAACC	2668	This study
	RR4-R	CTCAAAACTGAACAAAGTAAAGACG		
S10	S10-F	GACACGCTCGGACGCTTTGCCATGA	1708	This study
	S10-R	AATTCCATTATGCAAGCAACCTCCTC		

[a]RR1-RR4: four copies of 16S rRNA gene; S10: 30S ribosomal subunit protein S10 in *E. faecalis.*

Table S10. Primers for qRT-PCR in this study.

Primer	Primer sequence (5'-3')	Amplicon size (bp)
recA-F	CGACTAATGTCTCAAGCACTAC	106
recA-R	CGAACATCACGCCAACTT	
RS12140-F	ACACTAATCCACAACACAT	112
RS12140-R	TACTCTTGCTCAACTCTTC	
RS02205-F	TCTCAATGAACAAGCGGTAA	102
RS02205-R	GCCACGGTTAAGTTAGGAA	
RS06145-F	GCATCCAGATATTCAGGTA	195
RS06145-R	TTAGGCACAATAAGAACAAG	
RS11485-F	TTGCCTTAGTGTTAATCAT	107
RS11485-R	AACGGCTAATAGAAGAAC	
RS06880-F	GCATTAACAGAGATTCCT	179
RS06880-R	ATAATAACTTGACGACCTT	
RS00630-F	GTATTGGCTACTTGCTAA	136
RS00630-R	GCTGCTTCATTATCTCTAA	
RS11300-F	CTTGTGGTAACGGTAATG	113
RS11300-R	TAACGCTTCTTCTTGAAC	
RS10660-F	ACGAGCCAACAAATCATT	121
RS10660-R	TCAACCACTTCATCAACAA	
RS05865-F	ATTACCGACAGGCAACTT	134
RS05865-R	TTCCAGGTGCTAATAGATTCA	
RS09080-F	AGCAAGTTACGAGATGTA	200
RS09080-R	GTTGATAGGCACCATATAC	
RS12590-F	GGCATCAACCTTGGCTGTA	95
RS12590-R	GACTGGAATCAACGCAATCAC	
RS00350-F	CACCAGATAATGTCCAAT	94
RS00350-R	AAGAGCATAATTCCTAGTAA	

Table S11. Primers used for construction of gene overexpression strains.

Primer	Sequence (5'-3')	Product length (bp)	Underline (enzyme site)
**Construction of the overexpression plasmids**			
RS00630-F	CGCGGATCCCTATTTTTAGGAGGCTTTACAG	1152	BamHI
RS00630-R	CCGCTCGAGGGAACAATAATGAGCCCTTCT		XhoI
RS02205-F	TCCCCCGGGAAGAAGTGAAAGATGCGGTGA	1003	SmaI
RS02205-R	CCGCTCGAGAAGTTTCAATCAGTGCTTGCA		XhoI
RS06145-F	CGCGGATCCTGACTTGAGTTATGGCGGGTA	867	BamHI
RS06145-R	CCGCTCGAGAAGAATTGGTCGAACATCCAT		XhoI
RS06880-F	CGCGGATCCCCAAAACCCTTTACTCAGCGT	529	BamHI
RS06880-R	CCGCTCGAGAGCTTTCCGTTCAGGACGCTC		XhoI
RS11485-F	CGCGGATCCGTTGTCTTTTAAACGAGAGAG	962	BamHI
RS11485-R	CCGCTCGAGAAAGTATTCAACGCCCTGAAT		XhoI
RS12140-F	CGCGGATCCCATGAAGTTGCACCGAATCAT	1092	BamHI
RS12140-R	CCGCTCGAGATCACTTGCGGAATCATCAAT		XhoI
**Verification of the overexpression plasmids**			
IDRS00630-F	TTGACCGTGATCAAGATGCTG	624	
IDRS00630-R	AGACTGTAACATTCTCACGCA		
IDRS02205-F	AGTCGATACGCCAGAAGCCAT	623	
IDRS02205-R	GACTGTAACATTCTCACGCAT		
IDRS06145-F	AGCGAATGCAAGTGCAGAAGC	559	
IDRS06145-R	AGACTGTAACATTCTCACGCAT		
IDRS06880-F	TGCTTGGCGAAACGGTTTATG	604	
IDRS06880-R	ACTATTGCCGGGATAGACTGT		
IDRS11485-F	AGCAATAGGCATGGTAATTGT	524	
IDRS11485-R	ATGCGCCATGACAGCCATGAT		
IDRS12140-F	ATGTGTGACAACATTGCCATT	628	
IDRS12140-R	GACTGTAACATTCTCACGCAT		

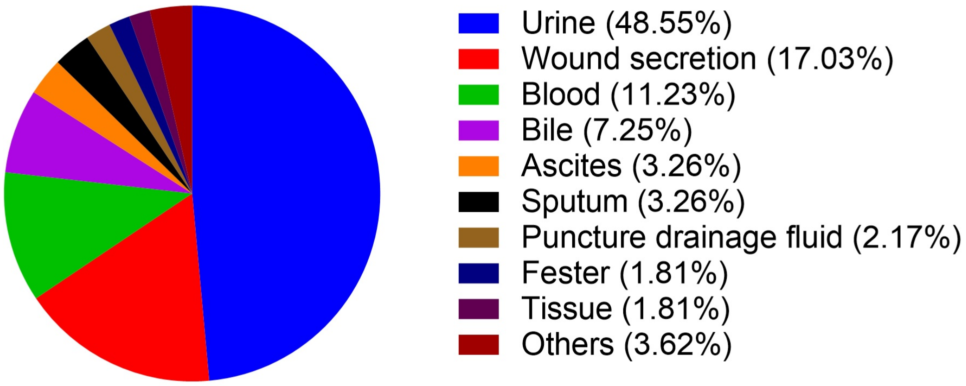

Figure S1. The original sources of 276 clinical *E. faecalis* isolates used in this study. Other sources included pleural effusion (3 strains), cerebrospinal fluid (2 strains), eye discharge (2 strains), vaginal discharge (2 strains), and amniotic fluid (1 strain).

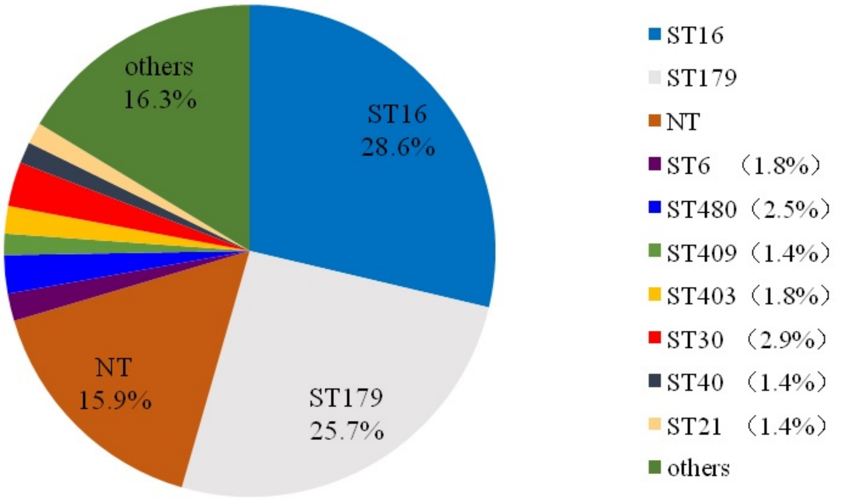

Figure S2. The distribution of multilocus sequence typing (MLST) among *E. faecalis*. Besides the number of STs described above, other MLST genotype included ST79 (1 strain), ST69 (1 strain), ST67 (1 strain), ST64 (1strain), ST63 (1 strain), ST585 (1 strain), ST581 (1 strain), ST541 (3 strains), ST506 (2 strains), ST483 (1 strain), ST474 (1 strain), ST47 (2 strains), ST436 (1 strain), ST410 (1 strain), ST41 (2 strains), ST4 (3 strains), ST387 (3 strains), ST34 (1 strain), ST314 (2 strains), ST300 (1 strain), ST28 (2 strains), ST22 (1 strain), ST207 (1strain), ST202 (1 strain), ST193 (3 strains), ST191 (1 strain), ST143 (1 strain), ST139 (1 strain), ST126 (1 strain), ST116 (1 strain), ST11 (1 strain), ST100 (1 strain). NT: not typeable.

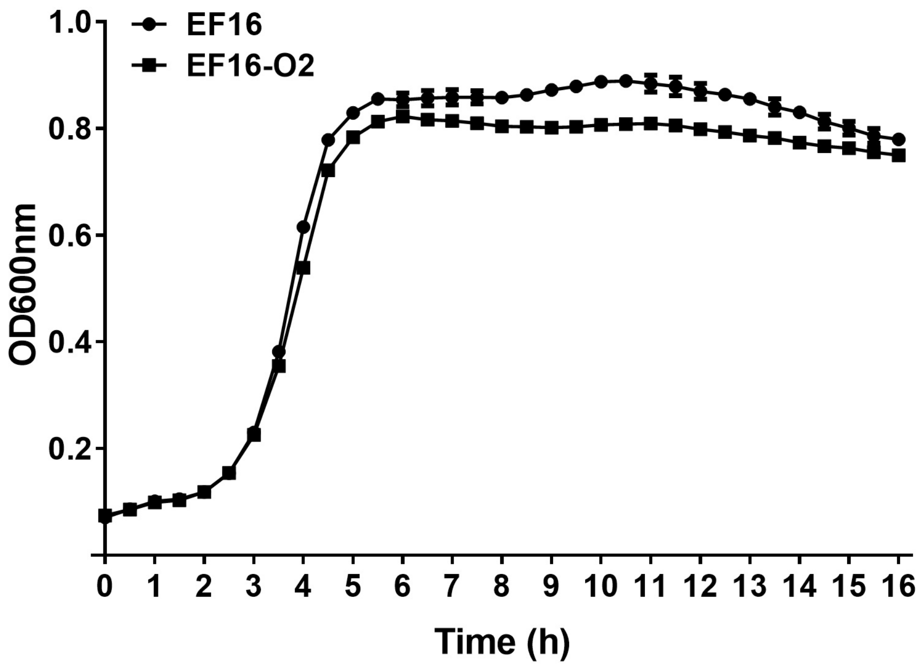

Figure S3. Growth curves of EF16C185 (EF16) and EF16C185-RS (EF16-O2) strains. Overnight bacterial cultures of EF16 or EF16-O2 were 1:200 diluted into MHB or MHB containing 1 mg/L OMC separately and incubated at 37℃ with shaking at 220 rpm. The OD600 was measured every 30 min. Values are means ± standard deviations from 3 independent wells.

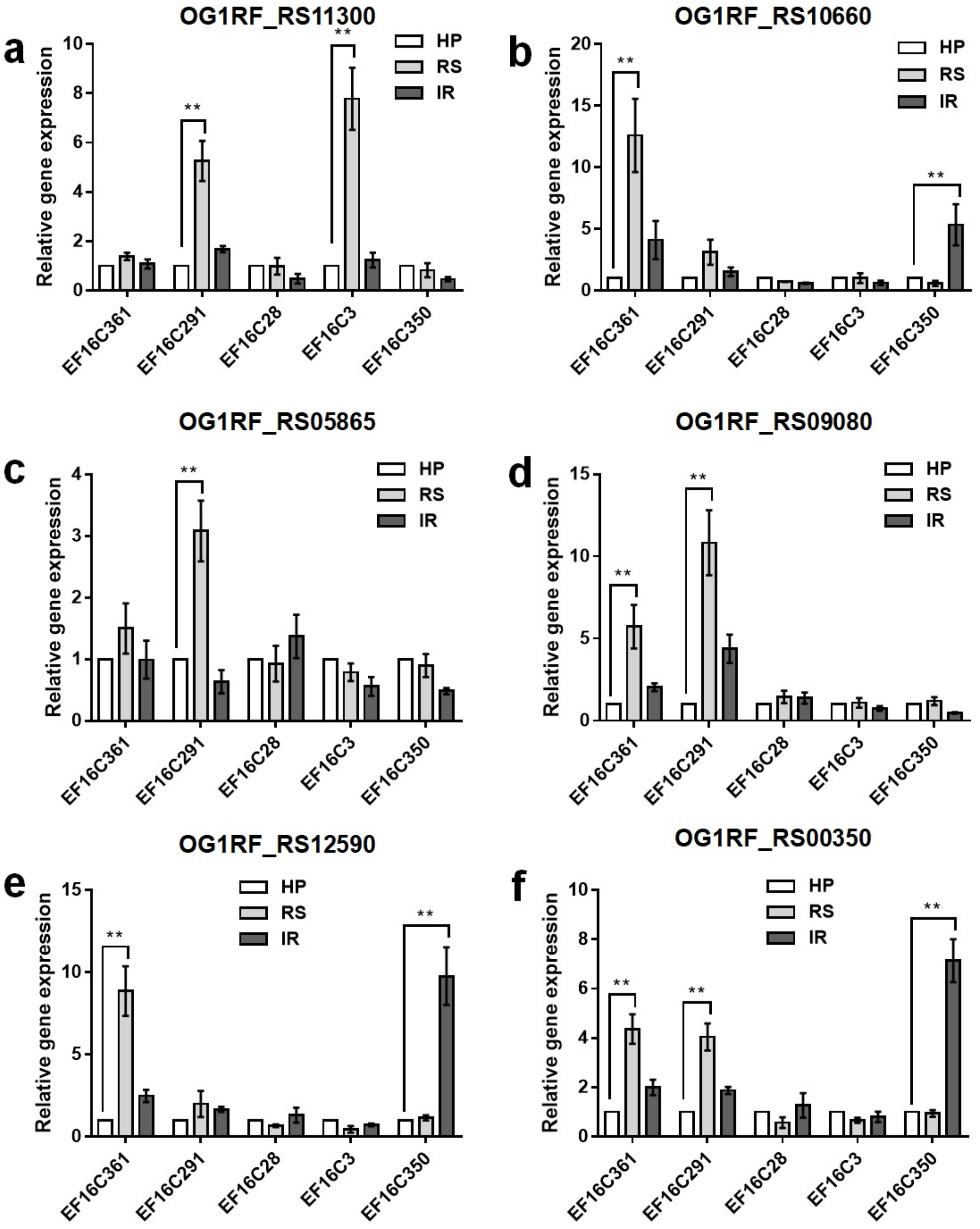

Figure S4. Comparison of the relative transcriptional analysis of six DEGs not used in overexpression experiments by qRT-PCR. Relative expressions of OG1RF_RS11300 (a), OG1RF_RS10660 (b), OG1RF_RS05865 (c), OG1RF_RS09080 (d), OG1RF_RS12590 (e), and OG1RF_RS00350 (f) were determined by qRT-PCR analysis. The housekeeping gene *recA* was used as the endogenous reference gene. The heteroresistant parental strain was used as the reference strain (expression = 1.0). All qRT-PCRs were carried out in triplicate with three independent RNA samples. HP: OMC heteroresistant parental strain; RS: OMC resistant subpopulations; IR, OMC-induced resistant strains. **: $p < 0.01$

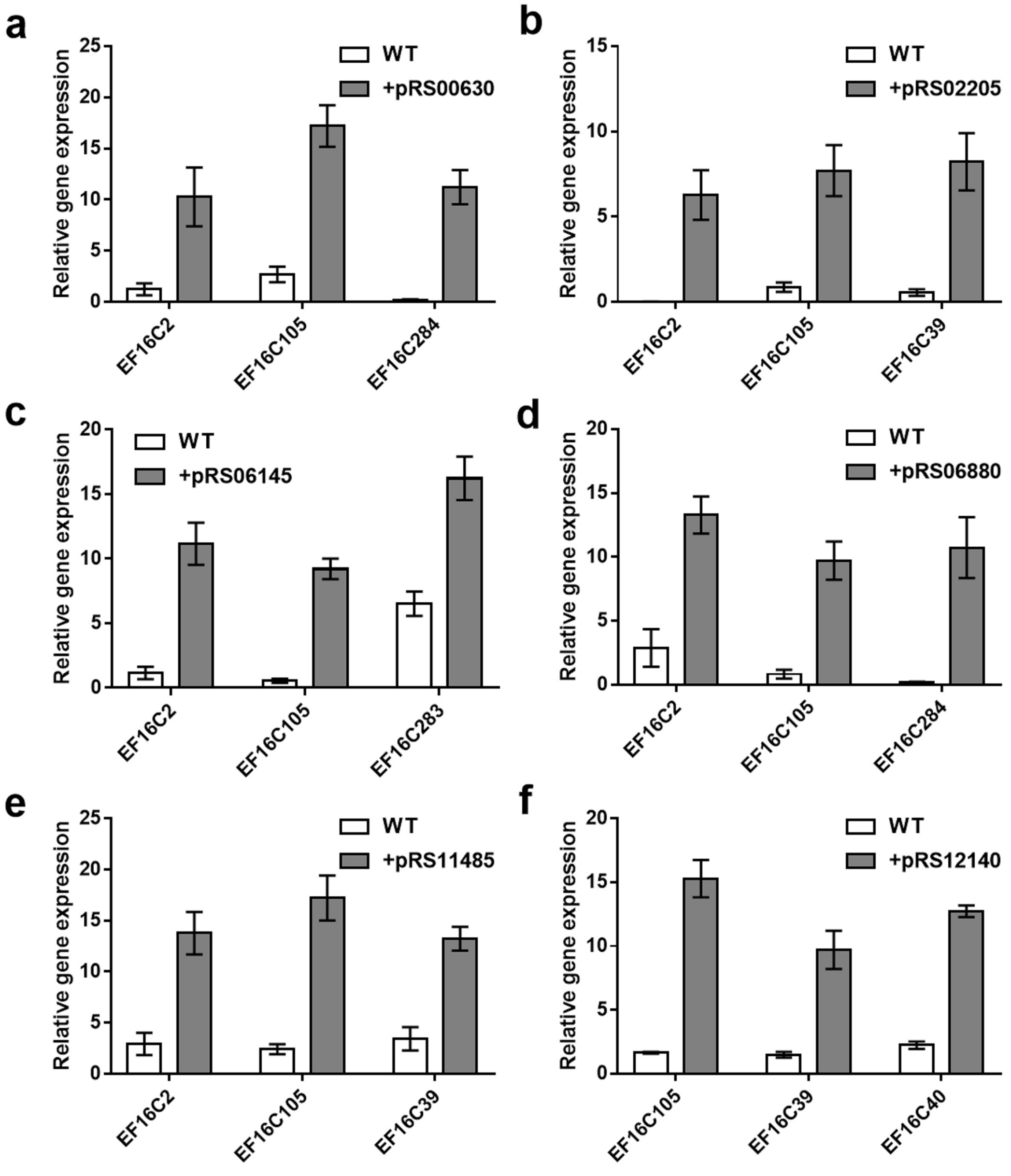

Figure S5. Relative transcriptional analysis of six candidate genes in different OMC-sensitive *E. faecalis* isolates and their corresponding transformed derivatives. The expression levels of OG1RF_RS00630 (a), OG1RF_RS02205 (b), OG1RF_RS06145 (c), OG1RF_RS06880 (d), OG1RF_RS11485 (e), and OG1RF_RS12140 (f) were determined by qRT-PCR. The housekeeping gene *recA* was used as the endogenous reference gene. The *E. faecalis* OG1RF strain was used as the reference strain (expression = 1.0). All qRT-PCRs were carried out in triplicate with three independent RNA samples.

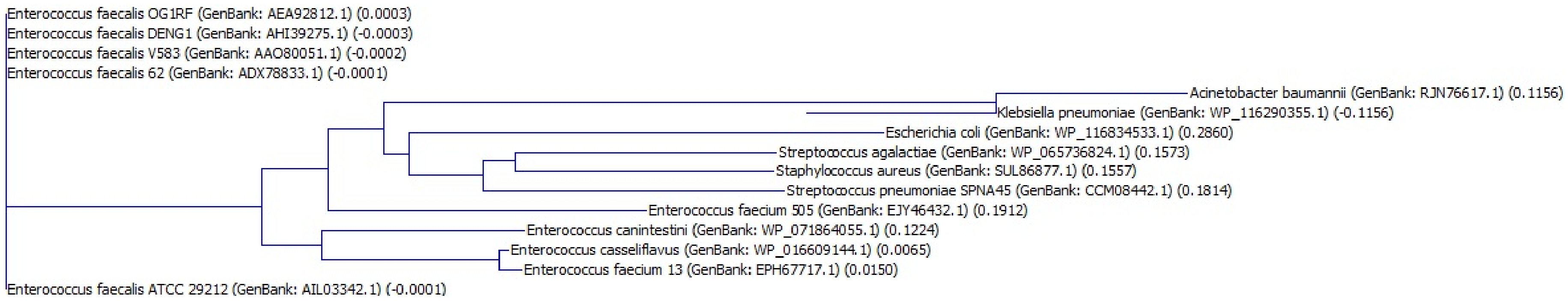

Figure S6. The phylogenetic tree of the BMP family ABC transporter substrate-binding protein encoded by OG1RF_RS00630.

References

1. Collins JR, Arredondo A, Roa A, Valdez Y, Leon R, Blanc V. 2016. Periodontal pathogens and tetracycline resistance genes in subgingival biofilm of periodontally healthy and diseased Dominican adults. Clin Oral Investig 20:349-56.
2. Nishimoto Y, Kobayashi N, Alam MM, Ishino M, Uehara N, Watanabe N. 2005. Analysis of the prevalence of tetracycline resistance genes in clinical isolates of Enterococcus faecalis and Enterococcus faecium in a Japanese hospital. Microb Drug Resist 11:146-53.
3. Ruiz-Garbajosa P, Bonten MJ, Robinson DA, Top J, Nallapareddy SR, Torres C, Coque TM, Canton R, Baquero F, Murray BE, del Campo R, Willems RJ. 2006. Multilocus sequence typing scheme for Enterococcus faecalis reveals hospital-adapted genetic complexes in a background of high rates of recombination. J Clin Microbiol 44:2220-8.
